# Multiverse: Multilingual Evidence for Fake News Detection †

**DOI:** 10.3390/jimaging9040077

**Published:** 2023-03-27

**Authors:** Daryna Dementieva, Mikhail Kuimov, Alexander Panchenko

**Affiliations:** 1 School of Computation, Information and Technology, Technical University of Munich, 80333 Munich, Germany; 2 Skolkovo Institute of Science and Technology, 121205 Moscow, Russia; 3 Artificial Intelligence Research Institute, 121108 Moscow, Russia

**Keywords:** fake news detection, multilinguality, news similarity, cross-lingual text similarity

## Abstract

The rapid spread of deceptive information on the internet can have severe and irreparable consequences. As a result, it is important to develop technology that can detect fake news. Although significant progress has been made in this area, current methods are limited because they focus only on one language and do not incorporate multilingual information. In this work, we propose Multiverse—a new feature based on multilingual evidence that can be used for fake news detection and improve existing approaches. Our hypothesis that cross-lingual evidence can be used as a feature for fake news detection is supported by manual experiments based on a set of true (legit) and fake news. Furthermore, we compared our fake news classification system based on the proposed feature with several baselines on two multi-domain datasets of general-topic news and one fake COVID-19 news dataset, showing that (in combination with linguistic features) it yields significant improvements over the baseline models, bringing additional useful signals to the classifier.

## 1. Introduction

The fast consumption of information from social media and news websites has become a daily routine for millions of users. Many readers neither have the time nor the interest (and/or skills) to fact-check every announced event. This opens up a wide range of opportunities to manipulate the opinions of citizens, one of which is fake news, which contains information about events that never happened in real life (or representations of real events in extremely narrow and biased ways). Fake news can be as simple as damaging the reputation of a person, organization, or country, or as serious as inciting immediate emotional reactions that lead to destructive actions in the physical world.

**Figure 1:**
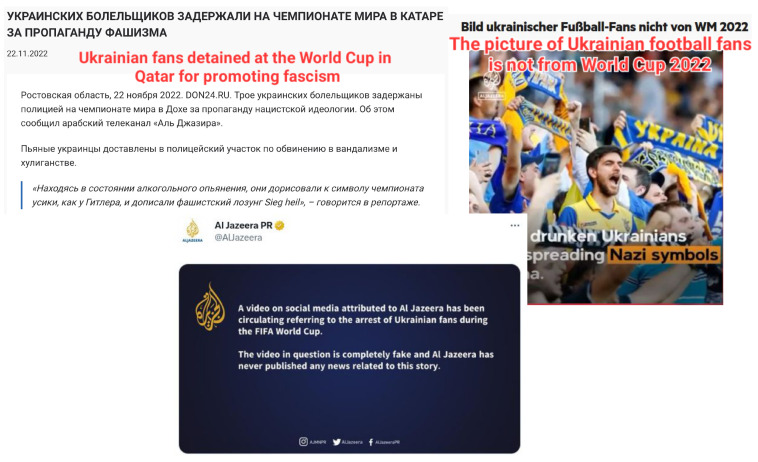
Example of the fake news spread of a video stated to be published by Al Jazeera about Ukrainian fans in Qatar (cf. image on **left**): e.g., see https://don24.ru/rubric/politika/ukrainskih-bolelschikov-zaderzhali-na-chempionate-mira-v-katare-za-propagandu-fashizma.html and https://twitter.com/MrPouquoi/status/1595487060068122628. This news item was fabricated both textually and visually. The described facts were refuted in several languages (cf. image on **right**) together with an official statement from the news source by Al Jazeera, e.g., see https://www.aljazeera.com/news/2022/11/24/fact-check-a-fake-video-of-ukrainian-nazi-fans, https://www.reuters.com/article/factcheck-al-jazeera-ukraine-idUSL1N32O0MR, and https://twitter.com/AlJazeera/status/1595779344198246401. This example illustrates the importance of news verification considering information from multilingual sources. All mentioned web pages were accessed on 14 December 2022.

Since the exploitation of Facebook to influence public opinion during the 2016 U.S. presidential election [1], there has been significant interest in fake news. However, the dissemination of false information not only misinforms readers but can also result in much more serious consequences. For instance, the spreading of a baseless rumor alleging that Hillary Clinton was involved in child sex trafficking led to a dangerous situation at a Washington D.C. pizzeria [2]. The global pandemic in 2020 has led to the rise of an infodemic [3], which could have even more severe consequences by exacerbating the epidemiological situation and endangering people’s health. Furthermore, The recent events of 2022 also showed how politics and global events could be dramatically influenced by the spread of fake news. The Russia–Ukraine conflict was accompanied by an intense information war [4] featuring an enormous amount of fake stories. In addition to the political world, the World Cup 2022 was surrounded by rumors from both organizers and visitors that had an impact on security during the competition [5]. A fake news story illustrated in Figure 1 is one artifact of this recent information warfare. Given these circumstances, it is more urgent than ever to develop technologies for reliable, open, and available-to-everyone fact-checking and news verification technologies.

The issue of fake news has garnered significant public attention and has also become a subject of growing interest in academic circles. With the proliferation of online content, there is a great deal of optimism about the potential of automated methods for detecting fake news. Numerous studies have been conducted on fake news detection, utilizing a variety of information from diverse sources. While the misinformation mitigation field is represented in the artificial intelligence field via different tasks (i.e., stance detection, fact-checking, source credibility classification, *inter alia*), we focus on the supervised fake news classification task.

For the fake news classification task, given a news *n* and its feature representation *F*, we aim to find a classifier f:(n,F)↦c that predicts class c∈C={Fake,Legit}. Discovering a suitable classifier entails searching and refining the classification model *f*, as well as extracting an appropriate set of feature representations *F*. Multiple supervised fake news detection models have been proposed based on linguistic features [6,7]; deep learning models [8,9,10,11]; or signals from social networks [12,13]. One direction of a supervised approach involves using additional information from the web [14,15,16]. In addition to text features, multimodal fake news detection has also been explored [17,18]. However, in these works only monolingual text signals were taken into account.

In our work, we propose a new way of how news feature representation *F* can be defined. We assume that viral spreading of (fake) information may naturally hit the “language barrier”; cross-checking of facts across media in various languages and cultures (that are supposed to be strongly independent) could yield additional signals. We aim to close the gap of monolingual textual feature usage; we explored cross-lingual web features for fake news detection.

The contributions of our work are as follows:

- We propose the ’Multiverse’: the new cross-lingual evidence feature for fake news detection based on multilingual news comparison. We explored several strategies for cross-lingual similarity estimations based on pre-trained neural language models.
- We conduct extensive experiments with the proposed feature showing its usefulness. First, a human judgment study is used for misinformation identification. Second, we integrate the feature into several fake news detection systems demonstrating that it consistently improves results and helps to improve performance.
- We demonstrate how our approach can be used to explain fake news detection to users by showing examples of how extracted cross-lingual information can be used for evidence generation.
- We demonstrate how the proposed technique can leverage image similarity by exploring two example news stories using image-based features.
- We made our implementation of the Multiverse feature and the code for our experiments openly available (https://github.com/s-nlp/multilingual-fake-news, accessed on 14 December 2022).

## 2. Related Work

For practical importance, fake news detection has become an active research topic in the field of natural language processing. A substantial amount of work has been conducted, including the creation of datasets and methods for automatic fake news identification. In this section, we perform a comprehensive analysis of prior art related to the subject of this article, structured into three parts: (1) the analysis of user behavior when attempting to fact check information, (2) fake news detection datasets, and (3) fake news detection methods.

### 2.1. User Behavior for Fake News Detection

Firstly, before the discussion of automatic machine fake news detection methods, we analyze how real-life users react to fake information and in which way they check the veracity of information.

In [19], a very broad analysis of users’ behavior was obtained. The authors discovered that when people attempt to check information credibility, they rely on a limited set of features, such as:

- Is this information compatible with other things that I believe to be true?
- Is this information internally coherent? Do the pieces form a plausible story?
- Does it come from a credible source?
- Do other people believe it?

Thus, people can rely on the news text, its source, and their judgment. However, if they receive enough internal motivation, they can also refer to some external sources for evidence. These external sources can be knowledgeable sources or other people.

The conclusions from [20] repeat the previous results: individuals rely on both their judgment of the source and the message. When these factors do not adequately provide a definitive answer, people turn to external resources to authenticate the news. The intentional and institutional reactions sought confirmation from institutional sources, with some respondents answering simply “Google”.

Moreover, several works have been conducted to explore the methods to combat fake information received by users and convince them with facts. In [21], it was shown that explicitly emphasizing the myth and even its repetition with refutation can help users pay attention and remember the truth. Additionally, participants who received messages across different media platforms [22] and different perspectives on the information [23] showed greater awareness of news evidence. Consequently, information obtained from external searches is an important feature for evaluating news authenticity and seeking evidence. Furthermore, obtaining different perspectives from different media sources adds more confidence decision-making process.

In our approach, we explore the use of this kind of evidence in the form of monolingual and cross-lingual news similarity scores.

### 2.2. Fake News Detection Datasets

To leverage the task of automatic fake news detection there have been created several news datasets focused on misinformation, each with a different strategy of labeling. The comparison of all discussed datasets is presented in Table 1.

The Fake News Challenge (http://www.fakenewschallenge.org, accessed on 14 December 2022) launched in 2016 was a big step in identifying fake news. The objective of FNC-1 was a stance detection task [24]. The dataset includes 300 topics, with 5–20 news articles each. In general, it consists of 50,000 labeled claim-article pairs. The dataset was derived from the Emergent project [25].

Another publicly available dataset is LIAR [26]. In this dataset 12,800 manually labeled short statements in various contexts from PolitiFact.com (*https://www.politifact.com*, accessed on 14 December 2022) were collected. They covered such topics as news releases, TV or radio interviews, campaign speeches, etc. The labels for news truthfulness are fine-grained in multiple classes: pants-fire, false, barely-true, half-true, mostly true, and true.

Claim verification is also related to the Fact Extraction and VERification dataset (FEVER) [27]; 185,445 claims were manually verified against the introductory sections of Wikipedia pages and classified as SUPPORTED, REFUTED, or NOTENOUGHINFO. For the first two classes, the annotators also recorded the sentences forming the necessary evidence for their judgments.

FakeNewsNet [28] contains two comprehensive datasets that include news content, social context, and dynamic information. Moreover, as opposed to all of the datasets described above, in addition to all of the textual information, there is also a visual component saved in this dataset. All news was collected via PolitiFact and GossipCop (https://www.gossipcop.com, accessed on 31 August 2021) crawlers. In general, 187,014 fake and 415,645 real news items were crawled.

Another dataset collected for supervised learning is the FakeNewsDataset [6]. The authors conducted a lot of manual work to collect and verify the data. As a result, they managed to collect 240 fake and 240 legit news items on 6 different domains—sports, business, entertainment, politics, technology, and education. All of the news articles in the dataset are from the year 2018.

One large dataset is NELA-GT-2018 [29]. In this dataset, the authors attempted to overcome some limitations that could be observed in previous works: (1) Engagement-driven—the majority of the datasets, for news articles and claims, contained only data that were highly engaged with on social media or received attention from fact-checking organizations; (2) lack of ground truth labels—all current large-scale news article datasets do not have any form of labeling for misinformation research. To overcome these limitations, they gathered a wide variety of news sources from varying levels of veracity and scraped article data from the gathered sources’ RSS feeds twice a day for 10 months in 2018. As a result, a new dataset was created consisting of 713,534 articles from 194 news and media producers.

**Table 1:** The datasets covered in related work. The majority of datasets for fake news detection tasks are in English.

Dataset	Task	Language
FNC-1 [24]	Stance Detection	English
Arabic Claims Dataset [30]		Arabic
FEVER [27]	Fact-Checking	English
DanFEVER [31]		Danish
LIAR [26]	Fake News Classification	English
FakeNewsNET [6]		
FakeNewsDataset [6]		
NELA-GT-2018 [29]		
ReCOVery [32]		
GermanFakeNC [33]		German
The Spanish Fake News Corpus [34]		Spanish

Due to the events of 2020, there has been ongoing work toward creating a COVID-19 fake news detection dataset. The COVID-19 Fake News [7] is based on information from public fact-verification websites and social media. It consists of 10,700 tweets (5600 real and 5100 fake posts) connected to the COVID-19 topic. In addition, the ReCOVery [32] multimodal dataset was created. It also incorporates 140,820 labeled tweets and 2029 news articles on coronavirus collected from reliable and unreliable resources.

However, all of the above datasets have one main limitation—they are monolingual and dedicated only to the English language. Regarding languages other than English, such datasets can be mentioned: *the French satiric dataset* [35], *GermanFakeNC* [33], *The Spanish Fake News Corpus* [34], and *Arabic Claims Dataset* [30]. These datasets do not fully cover the multilingualism gap in fake news detection. The mentioned datasets are monolingual as well and mostly cover fake news classification tasks, missing, for instance, fact verification and evidence generation problems.

In our work, we relied on English datasets for testing as they are the most commonly used and widespread for experiments. We studied how signals from other languages can improve the quality of monolingual fake news detection.

### 2.3. Fake News Classification Methods

Based on previously described datasets, multiple methods have been developed to tackle the problem of obtaining such a classifier. The feature sets used in all existing methods can be divided into two categories: (1) **internal** features that can be obtained from different preprocessing strategies and a linguistic analysis of the input text; (2) **external** features that are extracted from a knowledge base, the internet, or social networks, and give additional information about the facts from the news, its propagation in social media, and users’ reactions. In other words, internal methods rely on the text itself while external methods rely on meta-information from the text.

#### 2.3.1. Methods Based on Internal Features

Linguistic and psycholinguistic features are helpful in fake news classification tasks. In [6], a strong baseline model based on such a feature set was created based on the FakeNewsDataset. The set of features used in this work is as follows:

- **Ngrams**: tf–idf values of unigrams and bigrams from a bag-of-words representation of the input text.
- **Punctuation** such as periods, commas, dashes, question marks, and exclamation marks.
- **Psycholinguistic features** extracted with LIWC lexicon. Alongside some statistical information, LIWC also provides emotional and psychological analysis.
- **Readability** that estimates the complexity of a text. The authors use content features such as the number of characters, complex words, long words, the number of syllables, word types, and others. In addition, they used several readability metrics, including the Flesch–Kincaid, Flesch Reading Ease, Gunning Fog, and Automatic Readability Index.
- **Syntax** is a set of features derived from production rules based on context-free grammar (CFG) trees.

Using this feature set, the system yields strong results. That is why in our work we rely on it as a baseline, further extending this set with our newly developed features.

Based on such features, different statistical machine learning models can be trained. In [6], the authors trained the SVM classifier according to the set of characteristics presented. Naïve Bayes, Random Forest, KNN, and AdaBoost were also frequently used as fake news classification models [36,37,38].

In [39], the authors explore the potential of using emotional signals extracted from text to detect fake news. The authors analyzed the set of emotions present in true and fake news to test the hypothesis that trusted news sources do not use emotions to affect the reader’s opinion while fake news does. They discovered that emotions, such as *negative emotions*, *disgust*, and *surprise* tend to appear in fake news and can give a strong signal for fake news classification.

In addition to linguistic features, feature extraction strategies based on deep learning architectures were also explored. In [40], the classical architecture for the text classification task based on CNN was successfully applied to the fake news detection task. Given the recent surge in the use of Transformer architectures in natural language processing, models like BERT [10,41] and RoBERTa [9] have achieved high results in classifying general-topic fake news, as well as in detecting COVID-19-related fake news.

In addition to text features, images mentioned in news articles can serve as strong indicators for veracity identification. Visual content can be manipulated, for instance, via deepfakes [42] or by combining images from different contexts in a misleading format [43]. While multimodal fake news detection is a developing field, several approaches were already presented in [44,45].

It is evident that models based on internal feature sets have a significant advantage in their ease of use, as they do not require extensive additional time for feature extraction. Furthermore, such models can be highly efficient in terms of inference time and memory usage, as they solely rely on internal information from input news. However, if we take into account the aspect of explainability for end users, the evidence generated from such internal features is unlikely to be sufficient to persuade the user of the model’s accuracy and to justify the label assigned to the news.

#### 2.3.2. Methods Based on External Features

Although internal feature-based models can achieve high classification scores in the fake news classification task, the decisions of such are hard to interpret. As a result, additional signals from external sources can add more confidence to model decision reasoning.

If the news appears on a social network, information about the users who liked or reposted the item and the resulting propagation can serve as valuable features for fake news classification. It was shown in [46] that fake news tends to spread more quickly over social networks than true news. As a result, to combat fake news in the early stages of its appearance, several methods have been created to detect the anomaly behaviors in reposts or retweets [47,48]. In [49], different data about specific users were explored. The author extracted locations, profile images, and political biases to create a feature set.

User comments related to a news article can also serve as a valuable source of information for detecting fake news, and this approach was explored in [13]. The dEFEND system was created to explain fake news detection. The information from users’ comments was used to find related evidence and validate the facts from the original news. The Factual News Graph (FANG) system from [12] was presented to connect the content of news, news sources, and user interactions to create a fulfilled social picture of the inspected news.

To extract more information from the users who interact with news pieces, several approaches based on different word embeddings and transformer-based architectures can be applied. For instance, the SoulMate method [50] is specifically designed to measure short-text similarities, taking into account information about the authors. Moreover, we can take into account the personalities of the authors and predict their probability of spreading fake news, adapting the idea described in [51].

A simple source for obtaining evidence to verify the accuracy of information is the web. In several works, such as [14,15,16,52], the authors used web search engines, such as Google or Bing, to collect relevant articles; they also used scraped information, such as an external feature to build a fake news classifier. As discussed in Section 2.1, this web-based feature is motivated by the behaviors of real-life users. Consequently, the generated evidence based on scraped information can be more persuasive for users, as it automates the steps they typically take to verify the veracity of news articles.

However, in the discussed methods, we can also see the usage of only one language for evidence granting. The systems that used web search for evidence extraction turned to English search results only. In our work, we wanted to fill this gap to explore cross-lingual web-based evidence for the fake news classification task.

## 3. Multiverse: A New Feature for Fake News Classification

In this section, we present the general schema of our approach. We describe technical details of its implementation in two following sections. The Multiverse stands for Multilingual Evidence for Fake News Detection and is based on information extraction from a web search combined with cross-lingual text similarity and text categorization techniques. The idea of the approach is motivated by the user experience illustrated in Section 2.1 and the lack of multilingualism in automatic fake news detection methods, as discussed in Section 2.3. Users quite often refer to the web search to check news items seen in some news feeds. In order to overcome the limitations of a monolingual perspective and gain access to diverse viewpoints and supplementary information, conducting cross-lingual verification of news can be highly effective. Such an approach enables a broader scope for rational evaluation of information.

Our proposed approach is based on the following hypothesis:

**Hypothesis** **1** **(H1)** *If the news is true, then it will be widespread in the press, published in various languages, and published across media with different biases; the facts mentioned should also be identical. On the contrary, if the news is fake, it is likely to receive a lower response and be less widespread than a true news article, especially in foreign media. (Another reason that a true news article may not spread in foreign media is the fact that the mentioned event is just “too local” to be of interest to an international audience. To compensate for this obvious limitation, we take into account the ranks of the named entities mentioned in the news as described in Section 5.1.3).*

**Figure 2:**
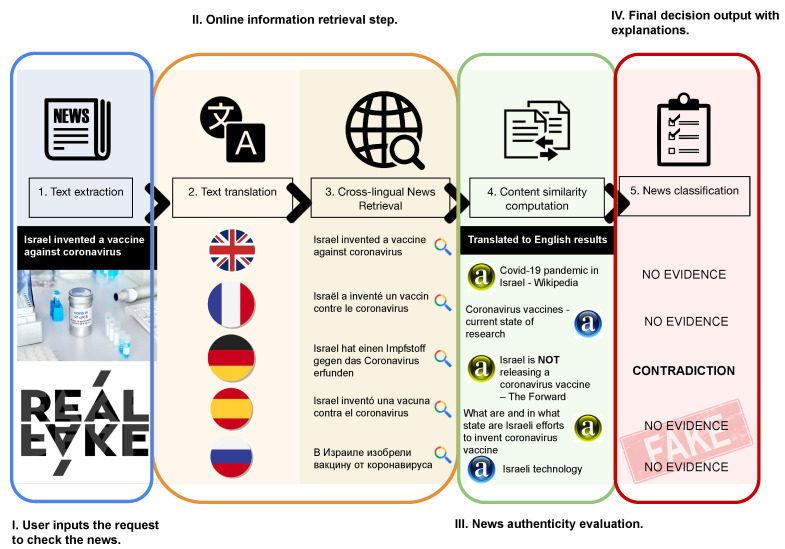
Overview of our approach: checking for fake news based on cross-lingual evidence (CE).

The step-by-step pipeline of the approach, schematically represented in Figure 2, is as follows:

- **Step 1. Text extraction:** As a new article arrives, the title and content are extracted from it. For instance, a user can generate such a request to check a piece of news.
- **Step 2. Text translation:** The title is translated into target languages and new search requests are generated. This is the preparatory step for multilingual news retrieval.
- **Step 3. Cross-lingual news retrieval:** Based on generated cross-lingual requests—translated titles—the search (via a web search engine) is executed. We suppose that this step should be accomplished with an online search available via a search engine.
- **Step 4. Cross-lingual evidence impacts computation:** Top-N articles from search results are extracted to assess the authenticity of the initial news. This step involves comparing the information presented in the news with that of the multilingual articles retrieved from a search query. The credibility and ranking of the news sources are also factored into the analysis. The objective is to estimate the number of articles that confirm or contradict the initial news.
- **Step 5. News classification:** Based on the information from the previous step, a decision is made about the authenticity of the news. If the majority of results support the original news, then it is more likely to be true; if there are contradictions—it is a signal to consider the news as fake.

As a result, a piece of news is represented with a feature set $F\left(n\right)={\left\{\left(s_{i}^{l_{j}},a_{i}^{l_{j}}\right)\right\}}_{i=1,j=1}^{i=N,j=L}$, where *N* stands for the number of news items extracted for each language, *L* is the number of languages used for cross-lingual news retrieval; *s* corresponds to the similarity score between two peace of news calculated with similarity metrics $\sigma :(n_{1},n_{2})\mapsto s$; *a* stands for the news source credibility score. The number of languages, scrapped news, as well as cross-lingual news similarity functions are hyperparameters of the proposed approach. In our work, we provide descriptions of how we define these features for our experiments.

From the example illustrated in Figure 2, we can see that for the news *“Israel invented a vaccine against coronavirus"*, the majority of the scraped articles provide no evidence that supported incoming news. Moreover, there was an article (originally, in German) with high reliability that provided an explicit refutation of the original information. As there is no supporting information and contradictions with the scraped information, the probability that we should believe in the veracity of the requested news is quite low.

The proposed method based on cross-lingual evidence extraction can work properly with important worldwide news. Indeed, if there is some local event about locally famous parties, in the majority of cases such news will doubtfully be widespread on the Internet. As a result, in our future assumptions and experiments, we will take into consideration datasets and news that cover worldwide events.

To confirm the hypothesis above, we conducted several experiments. For all experiments, we chose the top 5 European languages spoken in Europe (https://www.justlearn.com/blog/languages-spoken-in-europe, accessed on 20 March 2023) and used on the internet (https://www.statista.com/statistics/262946/share-of-the-most-common-languages-on-the-internet, accessed on 14 December 2022)—English, French, German, Spanish, and Russian—to obtain cross-lingual evidence. For the search engine, we stopped at Google Search (https://www.google.com, accessed on 14 December 2022) as it is the top search engine used in the world (https://www.oberlo.com/blog/top-search-engines-world, accessed on 14 December 2022) and was widely used by users during the fake news experiments mentioned in Section 2.1.

More specifically, we present the results of three experiments. The first experiment described in Section 4 is a manual small-scale study describing the methodology and experimental setup for manual news verification using Multiverse. In Section 5, we propose the methodology to automate the proposed pipeline. We describe how similarity functions can be estimated, source credibility rank can be extracted, and, finally, how the Multiverse feature set can be automatically constructed. Automated fake news detection systems were tested on several fake news classification datasets. We implemented our cross-lingual evidence feature and compared it with several baselines. The main difference between the first and the second experiment is the implementation of stages 4 and 5. In the manual setup, the estimation of cross-lingual news similarity and news categorization was done by human judges; in the automatic setup, we performed the similarity computations and categorization by automatically using machine learning models. The first three stages were conducted in both experiments similarly. In addition to two text-based experiments forming the core of our research, in Section 6, we deliver the third experiment by investigating the possibility of further extending the idea to the similarity of images retrieved using cross-lingual search.

## 4. Experiment 1: Manual Similarity Computation and Classification

To incorporate the proposed feature into an automatic fake news detection pipeline, firstly, we wanted to rely on the user experience; we checked the following hypothesis:

**Hypothesis** **2** **(H2)** *A person can detect fake news using the cross-lingual evidence feature as computed using the pipeline presented in Figure 2.*

To confirm Hypothesis 1 and 2, we experimented with manual markups where the annotators were asked to classify fake news based on cross-lingual evidence.

### 4.1. Dataset

For fake news examples, we used the top 50 fake news list from 2018, according to BuzzFeed (https://github.com/BuzzFeedNews/2018-12-fake-news-top-50, accessed on 14 December 2022). For true news, we used the NELA-GT-2018 dataset [29]. We manually selected 10 fake and true news items. We attempted to cover several topics in this dataset: celebrities, science, politics, culture, and the world. The full dataset featuring 20 news used for the manual markup is provided in Table 2.

### 4.2. Experimental Setup

As Google provides personalized search results (http://googlepress.blogspot.com/2004/03/google-introduces-personalized-search.html, accessed on 14 December 2022), we precomputed **Step 2** and **Step 3** for convenience and reproducibility. We generated cross-lingual requests in five languages—English, French, German, Spanish, and Russian. To translate from English, the Google Translation service was used. As the news items were from 2018, the time range of each search was limited to this year. For the cross-lingual search, the translated titles were used. From the search results, we used the first page of the search, which was composed of 10 news. As a result, for 20 news items for each language, we obtained 1000 pairs of “original news ↔ scraped news” to the markup.

**Figure 3:**
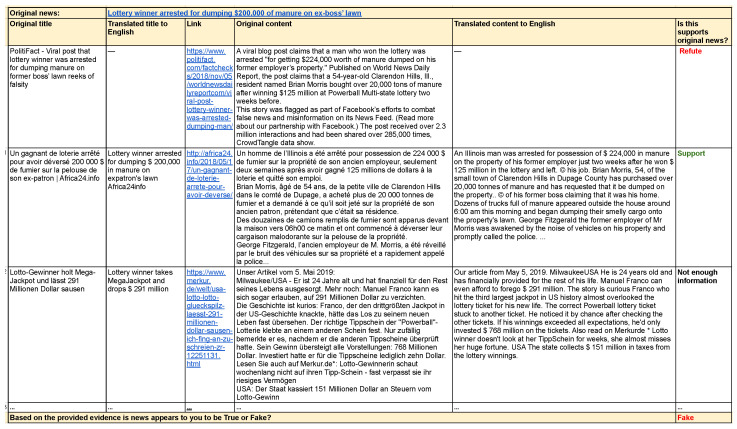
User interface that was used for the answer collection (for manual verification). The annotator was provided with original news and the link to the source. After that, the annotator was given the results of the cross-lingual search results translated into English if needed. For each news item from the search results, the title, link to the source, and text of the content were provided. The task assigned to the annotator was to determine whether the scraped news supported, refuted, or provided insufficient information to verify the original news. In the final step, the annotator was asked to classify the original news as either fake or true.

We asked six annotators to take part in the experiment, to manually conduct **Step 4**: cross-lingual evidence impact computation. For this, we created an interface for the markup presented in Figure 3 (a link to the original annotation table layout is available at https://github.com/s-nlp/multilingual-fake-news, accessed on 14 December 2022). For each news piece, we provide information about its title, content, and link to the source. As a result, every annotator could evaluate the quality of the text, the credibility of the source, and cross-lingual evidence for each sample from the dataset.

Every annotator received 10 randomly selected news items; as a result, each piece of news was cross-checked by 3 annotators. All non-English pieces of news were translated into English. For each pair, “original news ↔ scraped news”, the annotator provided one of three answers: (1) support: the information in the scraped news supports the original news; (2) refute: the information is opposite or differs from the original news or there is an explicit refutation; (3) not enough info: the information is not relevant or not sufficient enough to support/refute the original news. Finally, at the end of the annotation of a news item, the annotator was asked to proceed with **Step 5** of the pipeline and classify the news as fake or true.

### 4.3. Discussion of Results

**Figure 4:**
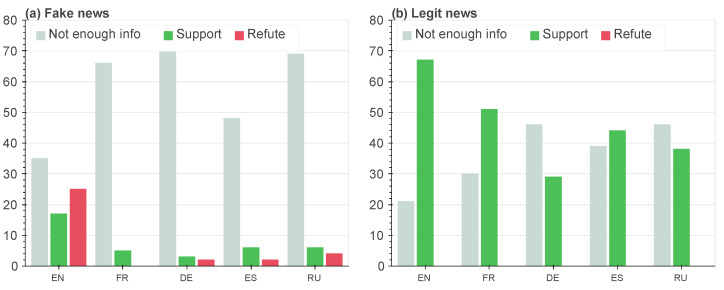
The results of the manual annotation: the distribution of answers for fake (**a**) and legit (**b**) news. As we can see, the amount of support news from the search results for every language for legit news incredibly overcomes the amount for fake news. At the same time, there is almost no ’refute’ news for legit news, while ’refute’ news appeared in the search results for fake news across all languages.

Based on the collected annotations, for each news item, we chose the final label based on the majority vote. We estimated the confidence in the annotator’s agreement with Krippendorff’s alpha (α=0.83). After that, we separately calculated the distribution of each answer for the top 10 search results by languages for fake and true news. The results are provided in Figure 4.

As we can see, the distribution of labels for true news significantly differs from the distribution for fake ones. The supporting articles are enough for almost every language. At the same time, we observed that for fake news, there were more signals refuting the English language version compared to supporting it. Furthermore, there was little to no evidence or relevant information dissemination in other languages. The obtained results can be used for the confirmation of Hypothesis 1. The fake news received less of a spread over different languages, while for true news, we can see supportive information from multilingual sources. Finally, the average accuracy of the annotator’s classification was 0.95. This confirms Hypothesis 2: a person can distinguish fake news based on cross-lingual evidence.

## 5. Experiment 2: Automatic Similarity Computation and Classification

In this section, we explore the possibilities to automate fake news classification using the cross-lingual evidence feature. Namely, we consider the following hypothesis:

**Hypothesis** **3** **(H3)** *The proposed cross-lingual evidence feature computed using the pipeline presented in Figure 2 can improve automatic fake news detection.*

We conducted chain experiments to validate this hypothesis by automating all of the steps of the pipeline presented in Section 3. We experimented with several approaches for cross-lingual evidence feature computation and compared the implementations with the annotator’s markup obtained in Section 4. After that, we incorporated our feature into an automated fake news detection pipeline by comparing it with baseline methods.

### 5.1. Automatic Cross-Lingual Evidence Feature

We implemented the cross-lingual evidence feature according to the steps of the pipeline described in Section 3. Firstly, we implemented Algorithms 1 and 2 to estimate cross-lingual texts similarity. Then, we implemented Algorithm 3, which automatically extracts cross-lingual evidence features for input news.
**Algorithm 1** News similarity evaluation using cosine distance.***Input***: news information *n*, web scraping result *w*, language of the search *l*.***Output***: similarity estimation for the news pair. 1:**function** cosine_distance_news_similarity(n,w,l) 2:     **if** type(w)isnottext **then** 3:         news_pair_similarity = 0 4:     **end if** 5:     **if** [l(“fake”),l(“false”),l(“lie”)]∈w **then** 6:          news_pair_similarity = 0 7:     **end if** 8:     news_pair_similarity=cosine_distance(mBERT(n),mBERT(w)) 9:     **return** news_pair_similarity10:**end function**

**Algorithm 2** News similarity evaluation using NLI.
***Input***: news information *n*, web scraping result *w*, language of the search *l*.
***Output***: similarity estimation for the news pair.
1:**function** nli_news_similarity(n,w,l)2:     news_pair_similarity=XNLI-RoBERTa(n,w)3:     **return** news_pair_similarity4:
**end function**



**Algorithm 3** Multilingual evidence for fake news detection: feature extraction.
***Input***: news information *n*, languages to use for comparison l∈L the maximum amount *N* of news from the web search to compare with.***Output***: cross-lingual evidence feature set $\left(s_{i},a_{i}\right)$ of similarity with the original news and source credibility rank for each news $w_{i}$ item from a multilingual web search.
 1:**function** Multiverse(*n*, *L*, *N*) 2:    cross_lingual_evidence := [] 3:    **for** l∈L **do** 4:         $headline_{l}=\mathrm{Translate}\left(n\left[headline\right],lang=l\right)$ 5:         $W=\mathrm{Search}(headline_{l},top=N)$ 6:         **for** w∈W **do** 7:             source_rank=AlexaRank(w) 8:             # For similarity score cosine- or NLI-based function can be chosen 9:             similarity=cross_lingual_news_similarity(n,w, l)10:             cross_lingual_evidence.append(similarity,source_rank)11:          **end for**12:      **end for**13:      **return** cross_lingual_evidence14:
**end function**



#### 5.1.1. Cross-Lingual Evidence Retrieval

To automate **Step 2**: *Text translation*, we used the Googletrans (https://pypi.org/project/googletrans, accessed on 20 March 2023) library. For the translation, we used five languages: English, French, German, Spanish, and Russian. To execute **Step 3**: *Cross-lingual News Retrieval*, the Google Search API (https://pypi.org/project/Google-Search-API, accessed on 20 March 2023) was used. As in the manual experiment, we generated queries by translating the titles of the original news and extracted only the first page of the search results, which gave us 10 articles for each language.

#### 5.1.2. Content Similarity Computation

The goal of **Step 4**: *Cross-lingual evidence impact computation* is to figure out if the information in scraped articles supports or refutes the information from the original news. To estimate the cross-lingual news similarity measurement σ, we tested two strategies: (1) similarity computation based on the cosine distance between text embeddings (Algorithm 1); (2) scores based on the NLI model (Algorithm 2).

##### Cosine Similarity

Firstly, we evaluated the similarity between two news items based on their text embeddings. As the similarity between text embeddings can be interpreted as the similarity between the text content, we assumed that such a strategy for content similarity computation can correlate with whether one news item supports information from another. However, there can be cases when the content of the news can be very close or even duplicated, but special remarks, such as “Fake”, “Rumor”, etc., indicate the refutation of the original facts. We took into account such situations. As a result, the algorithm for this content similarity computation approach is as follows:

1. If the link from the search leads to the file and not to the HTML page, then the news at this link is automatically considered dissimilar to the original one;
2. If there are signs of the disproof of news, such as the words “fake”, “false”, “rumor”, “lie” (and their translations to the corresponding language), negation, or rebuttal, then the news is automatically considered dissimilar to the original one;
3. Finally, we calculate the similarity between the news title and the translated original one. For the similarity measure, we choose the cosine similarity between sentence embeddings. To obtain the sentence vector representation, we average the sentence’s token embeddings extracted from multilingual Bert (mBERT) released by [53] (https://github.com/imgarylai/bert-embedding, accessed on 14 December 2022). If the similarity measure overcomes the threshold θ, then the data described in scraped news and original news are considered the same.

##### Natural Language Inference (NLI)

On the other hand, estimating the similarities between news items can be reformulated as the natural language inference task. NLI is the problem of determining whether a natural language hypothesis *h* can reasonably be inferred from a natural language premise *p* [54]. The relations between the hypothesis and premise can be *entailment*, *contradiction*, and *neutral*. The release of the large NLI dataset [55] and later multilingual XNLI dataset [56] makes the development of different deep learning systems to solve this task possible.

The number of classes and their meanings in the NLI task are very similar to the labels “Support”, “Refute”, and “Not enough info”, which are used for the stance detection task in the fake news detection pipeline and the manual markup. Moreover, in [57], the usage of NLI features for stance detection tasks was tested. The best model based on NLI features showed a 10% improvement in accuracy over baselines in the FNC-1 dataset. An example of the usage of the NLI model on news titles is presented in Table 3.

We used the XLM-RoBERTa-large model pre-trained on the multilingual XNLI dataset (https://huggingface.co/joeddav/xlm-roberta-large-xnli, accessed on 14 December 2022) to obtain NLI scores for the pairs “original news as premise *p*↔ scraped news as hypothesis *h*”. Moreover, we generated input in a special format: (1) the premise was formulated as “The news “<news title + first *N* symbols of content>” is legit”; (2) the hypothesis was only “<news title + first *N* symbols of content>”. The size *N* of the used content was a hyperparameter of this NLI-based approach for the news content similarity computation.

#### 5.1.3. Additional Features

##### Source Credibility

As discussed in Section 2.1, users often consider the credibility of the news source (*a*) when verifying the authenticity of a news piece. In addition, such a feature is widely used in the methods described in Section 2.3. In the Multiverse feature set *F*, we also take into account the credibility of the source from where the news comes from. Following [58], we used Alexa Rank for source assessment. More recent works, such as [59], further confirm the usefulness of Alexa-based metrics news media profiling.

##### Named Entity Frequency

During the manual experiment, it was discovered that a cross-lingual check is more relevant for news about worldwide important events, people, or organizations, and not the local ones. As a result, to evaluate the worthiness of the news to be checked in a cross-lingual manner, we (1) extracted NEs from the title and the content of the news with mBert fine-tuned for NER (https://huggingface.co/Babelscape/wikineural-multilingual-ner, accessed on 14 December 2022); (2) found the most relevant page on Wikipedia; (3) evaluated the Alexa Rank of the corresponding Wikipedia page to estimate the popularity of the NE. For our experiments, we assumed that all of the news items were about worldwide events. However, this additional feature should be included in the live fake news detection pipeline.

### 5.2. Comparison with Manual Markup

To assess the effectiveness of our chosen approaches for computing content similarity between news items, we conducted a small case study on a manually marked-up dataset. For each approach of news similarity estimation, we calculated the accuracy of such an experimental setup: the classification task if the scraped news supported the original news. The manually annotated dataset consisted of 1000 pairs of “original news ↔ scraped news”, each labeled by three annotators; the final label for each pair was determined by majority voting.

Taking this setup, we fine-tuned hyperparameters for both approaches. Specifically, we fine-tuned the threshold θ for the embedding-based similarity approach. We conducted a hyperparameter search on the [0.1,0.9] segment with δ=0.1. The best result was achieved with a θ value of 0.5 for decision-making on whether the scraped news supports the original news or not. For the NLI-based approach, we fine-tuned the length of the news text that was passed as input to the NLI model. We found that the optimal hyperparameters for this approach were a news text length of 500 symbols, which corresponds to the news title plus the first two paragraphs of the content. Additionally, we unified the “neutral” and “contradiction” classes in the NLI model to have a similar setup as the embeddings-based approach.

Finally, for the *cosine distance* approach, we achieved 82% accuracy, while for the *NLI* approach, we achieved 70% accuracy in the 1000-pairs dataset. Although the models are not ideal, we believe that they can be used as baseline approximations of human judgments.

### 5.3. Automatic Fake News Detection

Finally, we conducted a set of experiments to validate Hypothesis 3, i.e., if the presented cross-lingual evidence feature could improve automatic fake news detection systems. We integrated the automated cross-lingual evidence feature into the fake news classification pipeline tested on three datasets.

#### 5.3.1. Datasets

In the tested datasets for our automated experiment, we attempted to cover several worldwide topics—politics, famous people, events, entertainment, as well as the most recent events connected to COVID-19. Firstly, we evaluated the systems on a multi-domain dataset by [6], which consists of two parts: the *FakeNewsAMT* dataset (240 fake and 240 legit articles) and the *CelebrityDataset* dataset (250 fake and 250 legit articles). The *FakeNewsAMT* dataset consists of news from six topics: sports, business, entertainment, politics, technology, and education. *CelebrityDataset* is dedicated to rumors, hoaxes, and fake reports about famous actors, singers, socialites, and politicians. Secondly, we ran experiments on the COVID-19 fake news dataset *ReCOVery* [32]. It consists of 2029 (665 fake and 1364 true news). The full statistics about all used datasets is presented in Table 4. All datasets were originally in English.

We used the 70/20/10 proportion for the train/test/dev validation split.

#### 5.3.2. Baselines

We compared our approach with several baselines. For the baseline, we chose the fake news systems based on internal features computed either via linguistic analysis or neural networks.

**Linguistic Features**: In [6], a baseline fake news classification model was trained based on Ngrams, punctuation, psycholinguistic features extracted with LIWC, readability, and syntax. In [32], LIWC features were used as some of the proposed baselines. We tested these features separately, grouped them all, and in combination with our proposed feature. We experimented with SVM, RandomForest, LogRegression, and LightGBM. We used standard hyperparameters set for the models. The results of the best models based on LightGBM are presented. We refer to the model that combines all of the linguistic features mentioned above as the **All linguistic** model.

**Text-CNN, LSTM**: Following [32], we tested the classical model for text categorization of TextCNN and LSTM on all datasets.

**BERT, RoBERTa**: BERT [53]-based models were used for fake news detection by [10], specifically for COVID-19 fake news classification [9,11]. We used pre-trained models—BERT-based-uncased (https://huggingface.co/bert-base-uncased, accessed on 14 December 2022) and RoBERTa-based (https://huggingface.co/roberta-base, accessed on 14 December 2022)—and fine-tuned them.

**Only monolingual evidence (ME)**: In addition, we compared our feature with a baseline approach that only uses monolingual English evidence. In this case, we also utilized the LightGBM model.

#### 5.3.3. Results

To evaluate the performance of fake news classification models, we used three standard metrics: precision, recall, and $F_{1}$. The formulas are provided below:

(1) $$precision=\frac{TP}{TP+FP},recall=\frac{TP}{TP+FN},F_{1}=\frac{2\times precision\times recall}{precision+recall}$$

We experimented with both types of content similarity measurements—either cosine similarity between embeddings (Emb.) or NLI scores—concatenated with the source credibility rank (Rank) of the scraped news. Both Emb. and NLI features are presented as vectors of similarity scores for the pairs “original news ↔ scraped news”.

**Figure 5:**
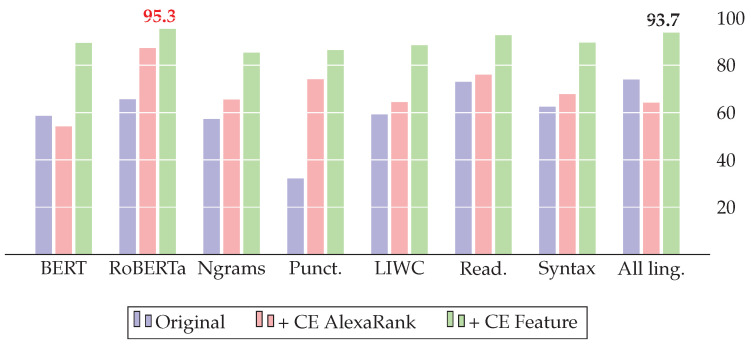
Results of the FakeNewsAMT dataset ($F_{1}$ score): adding the proposed cross-lingual evidence (CE) improves the various baseline systems and yields state-of-the-art results with the RoBERTa model.

**Figure 6:**
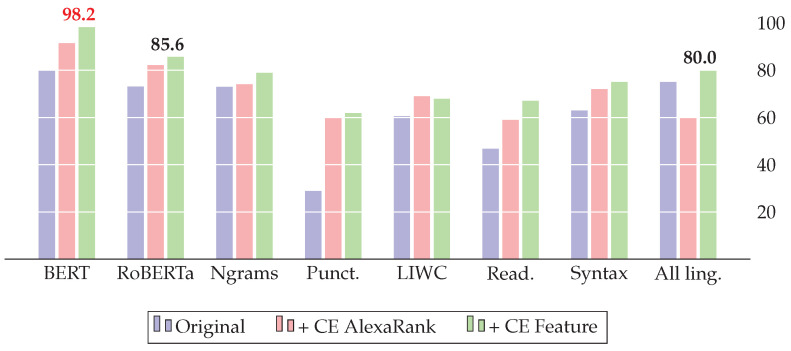
Results of the celebrity dataset ($F_{1}$ score): adding our cross-lingual evidence (CE) improves various baseline systems and yields state-of-the-art results with the BERT model.

**Figure 7:**
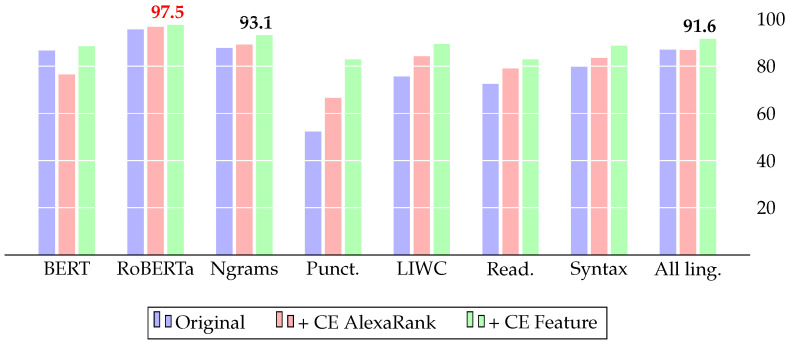
Results on the ReCOVert dataset ($F_{1}$ score): adding our cross-lingual evidence (CE) improves various baseline systems and yields state-of-the-art results with the RoBERTa model.

Table 5 compares the results of our model based on cross-lingual evidence (CE) with the baselines of three datasets. To prove the statistical significance of the results, we used the paired *t*-test on the five-fold cross-validation. All improvements presented in the results are statistically important. Additionally, we provide a histogram view of the $F_{1}$ score comparison for all three datasets: FakeNewsAMT (Figure 5), Celebrity (Figure 6), and ReCOVery (Figure 7).

The CE features exhibit slightly better performance than the baselines, or they perform comparably to the linguistic features. As such, a baseline fake news detection system can be built using only the CE feature, which offers greater explainability compared to other models. As expected, using only ME-based features for fake news detection yields worse results compared to using CE features. The NLI-based CE features generally exhibit worse results compared to the embeddings-based approach. To further improve performance, the NLI model can be specifically trained for the task of detecting confirmation or refutation in news content.

The addition of CE features improves all baseline models. For *FakeNewsAMT*, the best $F_{1}=0.973$ score is achieved with BERT embeddings in combination with CE features. For the *Celebrity* dataset, BERT with CE features shows the best results, achieving the best $F_{1}=982$ result. Despite RoBERTa showing the highest $F_{1}=0.975$ score for *ReCOVery*, the combination of all linguistic and CE features and specifically Ngrams with CE features shows competitive results, achieving $F_{1}=0.916$ and $F_{1}=0.931$, respectively.

**Figure 8:**
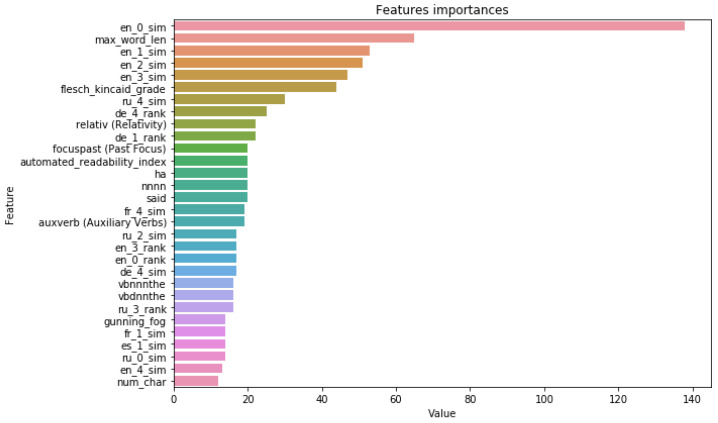
The top 30 feature importance scores of the best model for the FakeNewsAMT dataset: the LightGBM model based on all linguistic + CE Emb. + Rank feature set.

The importance of the proposed features in the model’s decision-making is also confirmed by the feature’s importance. The top 30 most important features for the best models for all datasets based on embeddings similarities are reported in Figure 8 and in Appendix A. For the *FakeNewsAMT*, *Celebrity*, and *ReCOVery* datasets, we can see the presence not only in English but in cross-lingual evidence features in the most important features. Although the English evidence features had the highest importance for the top three news from the search results, the similarity scores and rank of the source from other languages (such as French, German, Spanish, and Russian) also contributed to the overall performance.

### 5.4. Ablation Study

In order to verify which part of the presented cross-lingual evidence features impacted the results the most, we conducted an ablation study.

We compared the best results of the combinations of the linguistic features and CE evidence features. We tested the usage of monolingual English evidence (ME) and only source ranks (Rank) in combination with linguistic features. The results are presented in Table 6.

We can see that the rank of cross-lingual evidence sources compared to the combination of content similarity and ranks resulted in worse performance. This trend is also observed for the combinations of linguistic features with ME Rank (rank of only English sources), ME Emb. + Rank (content similarity comparison based on embeddings between the original news and only English search results in combination with English sources’ ranks), and CE Rank (the sources’ ranks of all scraped cross-lingual articles). For statistically significant proof for all comparisons with the best model, we used the paired *t*-test on the five-fold cross-validation. All of the obtained results are statistically significant. Consequently, we can claim that the use of proposed cross-lingual evidence features is justified.

### 5.5. Usage and Explainability

Our proposed feature can be easily utilized to explain the model’s decision and provide evidence from various sources confirming or refuting the verifiable news. For example, full-text evidence can be generated based on the obtained news to provide the user with at least the source of a critical attitude towards the original news. As the first step of such an explanation, we can report the scraped news, the rank of the source, and an approximation of the similarity to the original news. The examples of such a report are illustrated in Appendix B, Table A1 and Table A2. Finally, the proposed Multiverse feature can be framed into a platform for fake news detection. There, users can input the piece of news as a request and receive a cross-lingual news comparison. An example of such a demonstration can be found in Appendix C.

As can be seen from the examples, the hypothesis is confirmed by real pieces of news. For instance, in the case of the fake news example “Lottery winner arrested for dumping $200,000 of manure on ex-boss’ lawn”, we can see different scraped information. Some of the articles explicitly refute the news and name it as fake, e.g., the information from PolitiFact: “Viral post that lottery winner was arrested for dumping manure on former boss’ lawn reeks of falsity” and the Spanish list of fake news, i.e., “Estas son las 50 noticias falsas que tuvieron mayor éxito en Facebook en 2018”. However, some of the scraped news really copied the original title in different languages (“Un gagnant de loterie arrêté pour avoir déversé 200,000$ de fumier sur la pelouse de son ex-patron”, ПОБЕДИТЕЛЬ ЛОТЕРЕИ АРЕСТОВАН ЗА ТО, ЧТО ПОТРАТИЛ $200,000, ЧТОБЫ СВАЛИТЬ ГОРУ НАВОЗА НА ГАЗОН). However, we can see from the source ranks that these articles come from unreliable sources and the user should think critically about the information. The other titles either correlate the topic but give different information that does not support the original one (“Lotto-Gewinner holt Mega-Jackpot und lässt 291 Millionen Dollar sausen & Lottery winner takes MegaJackpot and drops”) or do not correlate in any way with the input article (“Histoire de Suresnes—Wikipedia & History of Suresnes—Wikipedia”). As a result, the user should be critical of the information since the confirmation number is quite small and there are even claims that the news is fake.

Contrary to the fake news, the legit news “Bubonic plague outbreak in Mongolia” received a large amount of support from all target languages. We can see the information about the bubonic plague is presented in the first cross-lingual results: “Bubonic plague: Case found in China’s Inner Mongolia”, “Epidémie : des cas de peste détectés en Chine et en Mongolie & Epidemic”, “Mongolei: 15-Jähriger an Beulenpest gestorben-DER SPIEGEL”, “BROTE DE PESTE BUBÓNICA EN MONGOLIA”, “В Мoнгoлии прoизoшла вспышка бубoннoй чумы”. Most importantly, the similarity between cross-lingual news content reinforces the fact that it comes from reliable sources. Thus, as we can see substantial cross-lingual support from trustworthy sources for the original news, the probability of believing in this information is quite high.

## 6. Experiment 3: Exploring the Use of Image Similarity Computation in the Multiverse

In this section, we present a preliminary study on further developing our method for the image domain. The idea in the following way:

1. The original headline is translated into 4 languages: English, French, German, Russian, and Spanish.
2. Requests via Google are made for each translated headline as well as the original one.
3. The top *k* relevant images are collected from the search results, where *k* is a hyperparameter of the approach.
4. Each image collected for each evidence language can be compared to each image collected from the search results for the headline in the original language to obtain pairwise image similarities.
5. Pairwise similarities are averaged to obtain the overall similarity of collected images.

**Figure A3:**
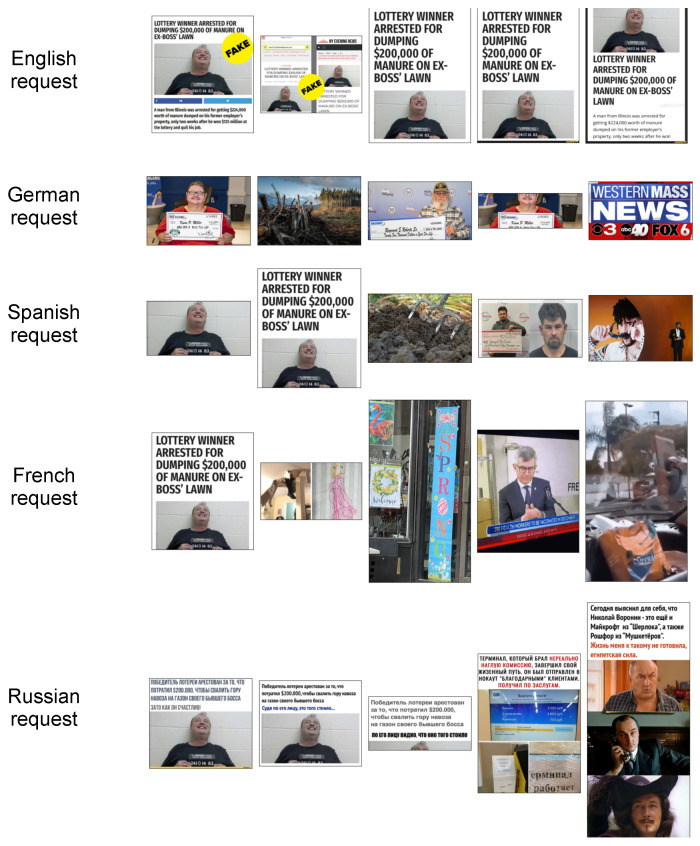
Top 5 images for fake news, “Lottery winner arrested for dumping $200,000 of manure on ex-boss’ lawn”.

**Figure A4:**
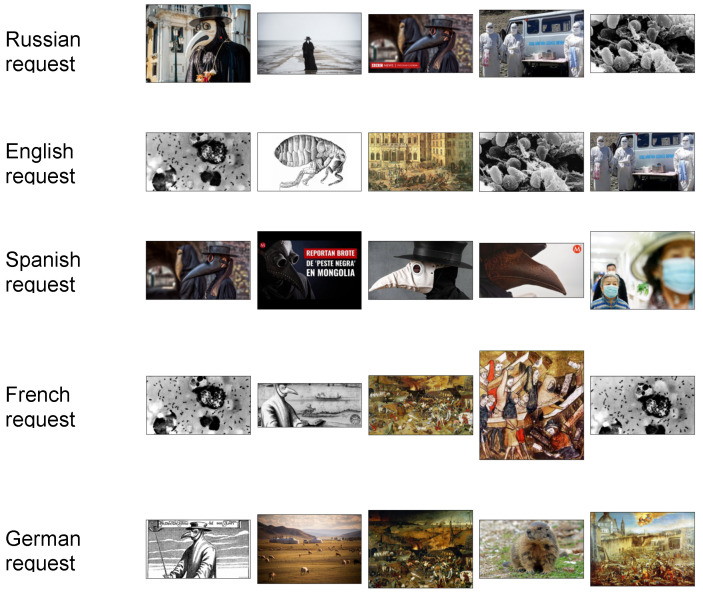
Top 5 images for legit news, “Bubonic plague outbreak in Mongolia”.

First, we need to note that this additional experiment was done to explore the overall possibility to transpose the idea to images. To illustrate our idea for future work, we picked two news examples from the appendix: one fake news item about the “lottery” and one legitimate news item about the “plague”. The examples are the same as those discussed in Section 5.5. Parameter *k* was set to 10. The top 5 collected images for each language are provided in Appendix B, Figure A3 and Figure A4. In order to compare images, the CLIP [60] model (we used the ViT-B/32 version of the model) was used for encoding. The resulting feature vectors were compared with the cosine similarity. Text for the model input was formed as “This is a picture for newsbreak ‘<news title>’”.

### Results

**Figure 9:**
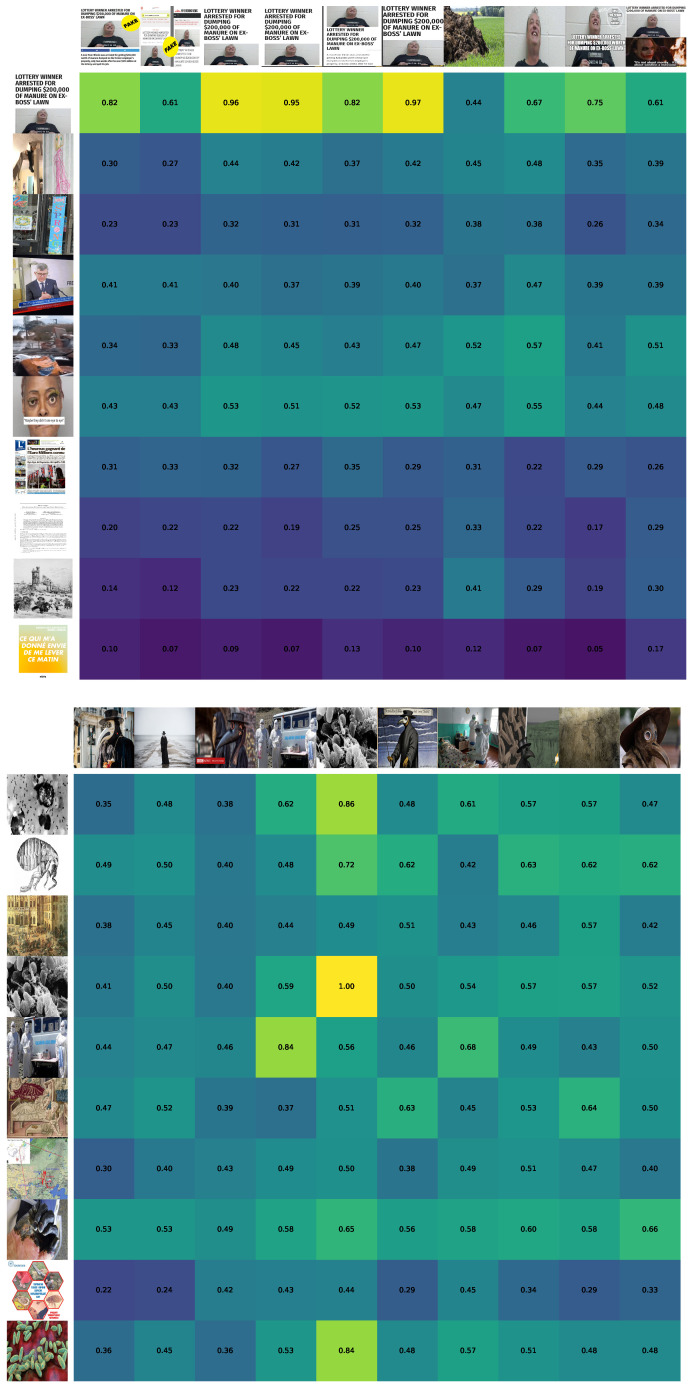
Examples of the pairwise image comparison with the cosine similarity for fake (**top**) and legit (**bottom**) pieces of news. The news corresponds to the two fake and true news items mentioned in the appendix, about, respectively, “lottery” and “plague”. At the **top** illustration, the heat map images from the English (**top** row of pictures) and French (**left** row of pictures) requests are compared. At the **bottom**—Russian and English images are compared.

The results of the image comparison for each language pair as well as the overall similarity are provided in Table 7. Moreover, we provide examples of the pairwise image comparison in Figure 9.

From Table 7, it can be assumed that for legit news the average similarity of images is higher than for fake news. However, such results could be the consequence of the fact that the selected legit piece of news is more general. More research is required for the hypothesis to be confirmed. Namely, a run on datasets used in Experiment 2 will be a reasonable setup.

## 7. Limitations and Future Work

The proposed cross-lingual evidence feature implemented using the described pipeline in Section 5.1 can have several limitations.

The main limitation is that our approach works best with globally important and widely spread events. Unfortunately, it is less applicable to local news related to specific regions or individuals. Nonetheless, even if the news concerns a well-known local figure, it is likely to be covered in news sources from neighboring countries. Consequently, a cross-lingual check with neighboring countries’ languages can also be useful.

Secondly, employing Google services for search and translation stages can introduce biases from personalized systems. To mitigate personalization in the search, we conducted experiments in incognito mode to conceal the search history and location parameters. However, Google Search can employ meta-information and tailor the resulting feed. On the one hand, using Google services is motivated by the user search experience. On the other hand, replicating such experiments can be challenging. To address this issue, one may use pre-saved snapshots of internet searches for a precise time period or use more anonymized search engines.

As we used automated translation to obtain the queries for cross-lingual search, there can be another side of such an automated translation application—some internet media can use automated translation to obtain the duplication of the news in the target language. Moreover, the methods for machine translation are becoming more advanced every year. As a result, we can obtain the repetition of the news in search results over the different languages. However, we believe that our proposed pipeline can handle such cases as we incorporated in our feature the source rank of the news. We believe that reliable media still self-process text material in their language. In future work, the addition of the detection of machine-generated text can be considered.

Another part that can be added to the proposed cross-lingual feature is the cross-checking of the information, not only with the original news but also between scraped multilingual evidence. That can add additional signals to the information verification process and reveal new details. Moreover, in this work, we used linguistic features calculated only for the original news. Such features can also be added to the all-scraped news in different languages when the appropriate methods are implemented.

In the presented experiments, the original news was presented only in English. Moreover, the datasets and information noise on the internet generally exist in larger amounts in English compared to any other language. In future work, it will make sense to test the proposed feature for the news originally presented in different languages other than English. Moreover, the amount of scraped evidence for language should be somehow normalized according to the overall amount of news that appears in the language.

## 8. Conclusions

We presented Multiverse—an approach for detecting fake news that utilizes cross-lingual evidence (CE). This approach is based on Hypothesis 1, which posits that news can propagate across languages, and is motivated by user behavior. The aim of the Multiverse is to overcome the constraints of previous work, which relied on only monolingual external features.

Firstly, we conducted a manual study on 20 news items to test Hypothesis 2, on whether the real-life user can use cross-lingual evidence to detect fake news. The annotators successfully passed the task of such news verification, providing the markup of 100 pairs “original news ↔ scraped news”. The annotators could detect fake news based on our proposed pipeline with an accuracy of 0.95, which confirms Hypothesis 2.

Then, we proposed Multiverse for automatic fake news detection to verify Hypothesis 3. To define the similarity measurement σ between cross-lingual news, we experimented with two strategies: (i) based on cosine distance between news text embeddings; (ii) based on natural language inference (NLI) scores, where the original news was used as the premise *p* and scraped news as the hypothesis *h*. We compared the proposed strategies with human assessments of 1000 pairs of marked news items, showing that these methods can be used for news similarity estimations. Finally, we integrated the proposed cross-lingual feature into the automated fake news detection pipeline. The cross-lingual feature itself showed the performance at the baseline level, proving a zero-shot usage of Multiverse to be a strong baseline fake news detection system. Moreover, the combination of our proposed feature with linguistic features based on the original news text yields significant classification results, outperforming both statistical and deep learning fake news classification systems. These results confirm Hypothesis 3: fake news detection systems benefit from Multiverse usage in both performance and explainability sides.

Additionally, we provided an ablation study, where the necessity of the usage of cross-lingual evidence with source rank, compared to only monolingual features was proven. We showed how our feature can be extended to visual content usage. Finally, to explore the explainability possibilities of Multiverse, we showed how the obtained cross-lingual information can be used for further evidence generation for the end users.

Finally, we presented a preliminary study on the integration of the similarity of visual illustrations of news with the proposed methodology, obtaining promising results worth further investigation.

## Figures and Tables

**Table 2:** The manually selected 20 news-item dataset (10 fake and 10 true news items) for the manual experiment. Fake news items were selected from the top 50 fake news items of 2018 according to BuzzFeed. Legit news items were selected from the NELA-GT-2018 dataset.

News Title	URL (All Webpages Were Accessed on 31 August 2021)	Label
Lottery winner arrested for dumping $200,000 of manure on ex-boss’ lawn	https://worldnewsdailyreport.com/lottery-winner-arrested-for-dumping-200000-of-manure-on-ex-boss-lawn/	Fake
Woman sues Samsung for $1.8 M after cell phone gets stuck inside her vagina	https://worldnewsdailyreport.com/woman-sues-samsung-for-1-8m-after-cell-phone-gets-stuck-inside-her-vagina/comment-page-58/	Fake
BREAKING: Michael Jordan Resigns from The Board at Nike-Takes ‘Air Jordans’ with Him	https://www.newsbreak.com/news/944830700924/breaking-michael-jordan-resigns-from-the-board-at-nike-takes-air-jordans-with-him	Fake
Donald Trump Ends School Shootings by Banning Schools	https://www.8shit.net/donald-trump-ends-school-shootings-banning-schools/	Fake
New mosquito species discovered that can get you pregnant with a single bite	https://thereisnews.com/new-mosquito-species-discovered-can-make-you-pregnant/	Fake
Obama Announces Bid to Become UN Secretary General	https://www.pinterest.com/pin/465630048969491948/	Fake
Lil Tay Rushed to Hospital after Being Beat by Group of Children at a Playground	https://www.huzlers.com/lil-tay-rushed-to-hospital-after-being-beat-by-group-of-children-at-a-playground/	Fake
Post Malone’s Tour Manager Quits Says Post Malone Smells Like Expired Milk And Moldy Cheese	https://www.huzlers.com/post-malones-tour-manager-quits-says-post-malone-smells-like-expired-milk-and-moldy-cheese/	Fake
Putin: Clinton Illegally Accepted $400 Million from Russia during Election	https://newspunch.com/putin-clinton-campaign-400-million-russia/	Fake
Elon Musk: 99.9% of Media Is Owned by the ‘New World Order’	https://newspunch.com/elon-musk-media-owned-new-world-order/	Fake
Scientists Develop New Method to Create Stem Cells without Killing Human Embryos	https://www.christianpost.com/news/scientists-develop-new-method-to-create-stem-cells-without-killing-human-embryos.html	Legit
Luis Palau Diagnosed with Stage 4 Lung Cancer	https://cnnw.com/luis-palau-diagnosed-with-stage-4-lung-cancer/	Legit
1st black woman nominated to be Marine brigadier general	https://edition.cnn.com/2018/04/12/politics/marine-corps-brigadier-general-first-black-female/index.html	Legit
Disney CEO Bob Iger revealed that he seriously explored running for president	https://www.businessinsider.com/disney-ceo-bob-iger-says-he-considered-running-for-president-oprah-pushed-2018-4	Legit
Trump Has Canceled via Twitter His G20 Meeting with Vladimir Putin	https://www.buzzfeednews.com/article/emilytamkin/trump-g20-putin-russia	Legit
US Mexico and Canada sign new USMCA trade deal	https://www.dw.com/en/us-mexico-canada-sign-usmca-trade-deal/a-51613992	Legit
Afghanistan Women children among 23 killed in US attack UN	https://www.aljazeera.com/news/2018/11/30/afghanistan-women-children-among-23-killed-in-us-attack-un	Legit
UNESCO adds reggae music to global cultural heritage list	https://www.aljazeera.com/features/2018/11/29/unesco-adds-reggae-music-to-global-cultural-heritage-list	Legit
The Saudi women detained for demanding basic human rights	https://www.aljazeera.com/news/2018/11/29/the-saudi-women-detained-for-demanding-basic-human-rights/	Legit
Georgia ruling party candidate Zurabishvili wins presidential runoff	https://www.aljazeera.com/news/2018/11/30/ex-envoy-wins-georgia-presidency-vote-to-be-challenged	Legit

**Table 3:** Example of how the NLI model can be used to extract relations between news.

Premise *p*	Hypothesis *h*	Label
Israel invented a vaccine against coronavirus	Israel is not releasing a coronavirus vaccine—The Forward	contradiction
Israel invented a vaccine against coronavirus	COVID-19 pandemic in Israel—Wikipedia	neutral
Israel invented a vaccine against coronavirus	Israel’s vaccine has 90% efficacy in trial	entailment

**Table 4:** Statistics of the datasets that were used to test fake news classification with the proposed Multiverse feature.

Dataset	# Fakes	# Legit	Covered Topics
FakeNewsAMT	240	240	sports, business, entertainment, politics, technology, and education
CelebrityDataset	250	250	rumors, hoaxes, and fake reports about famous actors, singers, socialites, and politicians
ReCOVery	665	1364	rumors, hoaxes, and fake news about COVID-19

**Table 5:** Results of the integration of the cross-lingual evidence (CE) feature into automated fake news classification systems. The proposed feature is used in two ways based on the content similarity computation strategy: (i) based on text embeddings (Emb.); (ii) based on NLI scores (NLI). It is also combined with the rank of the news article source (Rank). The CE feature along already showed the results better then the baseline methods. However, in combination with linguistic features, the SOTA results were achieved. The **bold** numbers indicates the best results in the block, the **bold and underlined**—the best result for the dataset. All improvements in the results were statistically proven by the *t*-test on the 5-fold cross-validation.

	FakeNewsAMT			Celebrity			ReCOVery		
	Pre.	Rec.	F1	Pre.	Rec.	F1	Pre.	Rec.	F1
TextCNN	0.276	0.250	0.260	0.641	0.703	0.664	0.733	0.913	0.805
LSTM	0.614	0.614	0.614	0.745	0.740	0.740	0.800	0.803	0.793
ME Emb. + Rank	0.539	0.593	0.592	0.552	0.550	0.550	0.794	0.798	0.793
ME NLI + Rank	0.637	0.633	0.634	0.554	0.550	0.550	0.756	0.761	0.752
CE Emb. + Rank	**0.872**	**0.864**	**0.864**	**0.631**	**0.620**	0.619	**0.829**	**0.829**	**0.829**
CE NLI + Rank	0.837	0.833	0.834	0.625	**0.620**	**0.620**	0.767	0.771	0.762
BERT	0.586	0.586	0.586	0.800	0.800	0.800	0.868	0.868	0.866
BERT + CE Emb + Rank	**0.884**	**0.885**	**0.894**	**0.982**	**0.982**	**0.982**	**0.870**	**0.863**	**0.884**
RoBERTa	0.895	0.548	0.656	0.856	0.690	0.731	0.986	0.936	0.956
RoBERTa + CE Emb + Rank	**0.973**	**0.938**	**0.953**	**0.952**	**0.784**	**0.856**	**0.992**	**0.960**	**0.975**
Ngrams	0.573	0.572	0.572	0.730	0.730	0.730	0.878	0.879	0.877
Ngrams + CE Emb. + Rank	**0.864**	**0.854**	**0.853**	**0.789**	**0.790**	**0.789**	**0.931**	**0.932**	**0.931**
Ngrams + CE NLI + Rank	0.844	0.844	0.844	0.690	0.690	0.690	0.862	0.860	0.856
Punctuation	0.239	0.489	0.321	0.211	0.460	0.289	0.433	0.658	0.522
Punctuation + CE Emb. + Rank	**0.872**	0.864	0.864	0.631	0.620	0.619	**0.829**	**0.829**	**0.829**
Punctuation + CE NLI + Rank	0.870	**0.865**	**0.865**	**0.690**	**0.690**	**0.690**	0.767	0.771	0.762
LIWC	0.597	0.593	0.592	0.630	0.610	0.605	0.768	0.771	0.756
LIWC + CE Emb. + Rank	**0.894**	**0.885**	**0.884**	**0.692**	**0.680**	**0.679**	**0.894**	**0.894**	**0.894**
LIWC + CE NLI + Rank	0.850	0.844	0.844	0.650	0.650	0.650	0.816	0.815	0.808
Readability	0.729	0.729	0.729	0.478	0.470	0.468	0.732	0.741	0.724
Readability + CE Emb.+ Rank	**0.928**	**0.927**	**0.927**	**0.674**	**0.670**	**0.670**	**0.828**	**0.829**	**0.828**
Readability + CE NLI + Rank	0.854	0.854	0.854	0.601	0.600	0.599	0.772	0.773	0.762
Syntax	0.626	0.625	0.624	0.639	0.630	0.629	0.812	0.809	0.797
Syntax + CE Emb. + Rank	**0.902**	**0.895**	**0.895**	**0.754**	**0.750**	**0.750**	**0.886**	**0.886**	**0.886**
Syntax + CE NLI + Rank	0.505	0.500	0.501	0.525	0.520	0.519	0.840	0.837	0.832
All linguistic	0.739	0.739	0.739	0.750	0.750	0.750	0.875	0.874	0.870
All linguistic + CE Emb. + Rank	**0.940**	**0.937**	**0.937**	**0.801**	**0.800**	**0.800**	**0.916**	**0.917**	**0.916**
All linguistic + CE NLI + Rank	0.886	0.885	0.886	0.737	0.732	0.732	0.864	0.865	0.862

**Table 6:** Results of the ablation study—the use of the best feature sets with cross-lingual evidence (CE) and source rank (Rank) compared with the use of monolingual evidence (ME) and the source rank. The **bold** numbers indicates the best results in the block, the **bold and underlined**—the best result for the dataset. We can see that the performances of only source ranks or monolingual evidence are significantly worse than the use of our proposed feature for all datasets.

	FakeNewsAMT			Celebrity			ReCOVery		
	Pre.	Rec.	F1	Pre.	Rec.	F1	Pre.	Rec.	F1
CE Rank	0.541	0.541	0.541	0.605	0.605	0.605	0.768	0.773	0.765
CE Emb. + Rank	**0.872**	**0.864**	**0.864**	**0.631**	**0.620**	**0.619**	**0.829**	**0.829**	**0.829**
Ngrams + ME Rank	0.646	0.645	0.644	0.679	0.680	0.679	0.802	0.802	0.800
Ngrams + ME Emb. + Rank	0.656	0.656	0.656	0.750	0.750	0.750	0.808	0.807	0.805
Ngrams + CE Rank	0.655	0.655	0.655	0.740	0.740	0.740	0.891	0.891	0.891
Ngrams + CE Emb. + Rank	**0.864**	**0.854**	**0.853**	**0.789**	**0.790**	**0.789**	**0.931**	**0.932**	**0.931**
Punct. + ME Rank	0.604	0.604	0.603	0.589	0.590	0.589	0.718	0.721	0.717
Punct. + ME NLI + Rank	0.855	0.854	0.854	0.670	0.670	0.670	0.756	0.761	0.752
Punct. + CE Rank	0.741	0.741	0.741	0.605	0.600	0.600	0.668	0.673	0.665
Punct. + CE NLI + Rank	**0.870**	**0.865**	**0.865**	**0.690**	**0.690**	**0.690**	0.**767**	**0.771**	**0.762**
LIWC + ME Rank	0.646	0.645	0.643	0.617	0.610	0.610	0.771	0.771	0.769
LIWC + ME Emb. + Rank	0.713	0.708	0.705	0.643	0.640	0.640	0.850	0.851	0.848
LIWC + CE Rank	0.646	0.645	0.644	0.712	0.700	0.690	0.846	0.846	0.842
LIWC + CE Emb. + Rank	**0.894**	**0.885**	**0.884**	**0.692**	**0.680**	**0.679**	**0.894**	**0.894**	**0.894**
Read. + ME Rank	0.650	0.650	0.650	0.530	0.530	0.530	0.797	0.801	0.796
Read. + ME Emb. + Rank	0.739	0.739	0.739	0.580	0.580	0.580	0.808	0.811	0.806
Read. + CE Rank	0.760	0.760	0.760	0.592	0.590	0.590	0.796	0.798	0.790
Read. + CE Emb. + Rank	**0.928**	**0.927**	**0.927**	**0.674**	**0.670**	**0.670**	**0.828**	**0.829**	**0.828**
Syntax + ME Rank	0.670	0.666	0.663	0.620	0.620	0.620	0.754	0.754	0.749
Syntax + ME Emb. + Rank	0.689	0.677	0.670	0.656	0.650	0.650	0.806	0.805	0.805
Syntax + CE Rank	0.677	0.677	0.677	0.721	0.720	0.720	0.844	0.841	0.834
Syntax + CE Emb. + Rank	**0.902**	**0.895**	**0.895**	**0.754**	**0.750**	**0.750**	**0.886**	**0.886**	**0.886**
All ling. + ME Rank	0.604	0.604	0.603	0.589	0.590	0.589	0.808	0.807	0.804
All ling. + ME Emb. + Rank	0.803	0.802	0.801	0.759	0.760	0.759	0.808	0.807	0.804
All ling. + CE Rank	0.641	0.641	0.641	0.605	0.600	0.600	0.868	0.868	0.868
All ling. + CE Emb. + Rank	**0.940**	**0.937**	**0.937**	**0.801**	**0.800**	**0.800**	**0.916**	**0.917**	**0.916**

**Table 7:** Results of the cross-lingual image comparison: “anecdotal evidence” suggesting that this line of work may be worth further exploration.

Title in English	Original Language	Evidence Language	Similarity Score
FAKE news			
*Lottery winner arrested for dumping*	en	ru	0.47
*$200,000 of manure on ex-boss’ lawn.*	en	fr	0.37
	en	es	0.44
	en	de	0.36
**Overall similarity**			0.41
LEGIT news			
*Bubonic plague outbreak in Mongolia.*	ru	en	0.50
	ru	fr	0.51
	ru	es	0.53
	ru	de	0.51
**Overall similarity**			0.51

**Figure A1:**
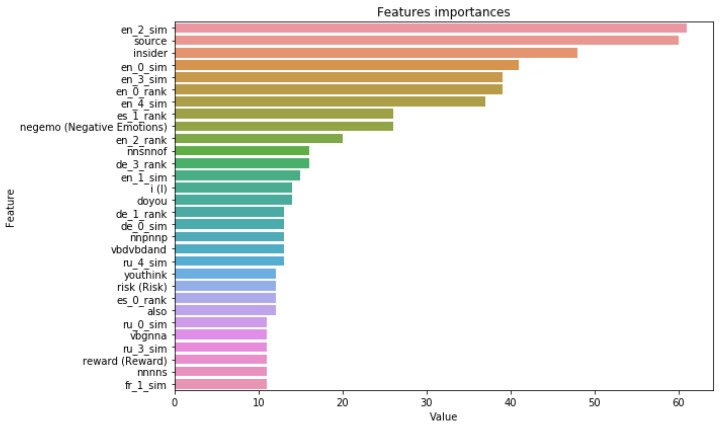
Top 30 feature importances of the best model for the celebrity dataset: LightGBM model based on all linguistic + CE Emb. + Rank feature set.

**Figure A2:**
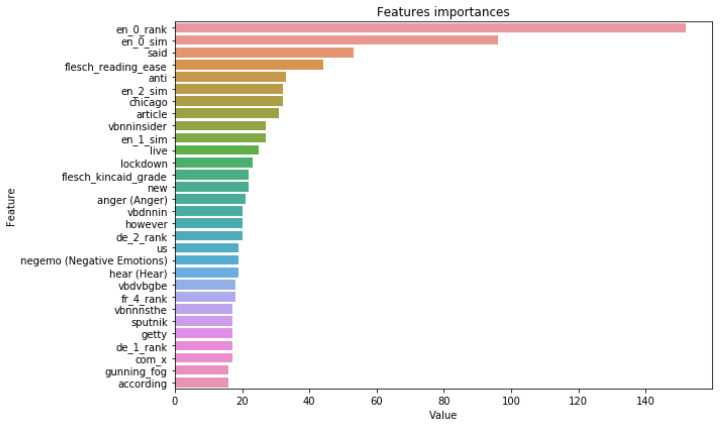
Top 30 feature importances of the best model for the ReCOVery dataset: LightGBM model based on Ngrams + CE Emb. + Rank feature set.

**Figure A5:**
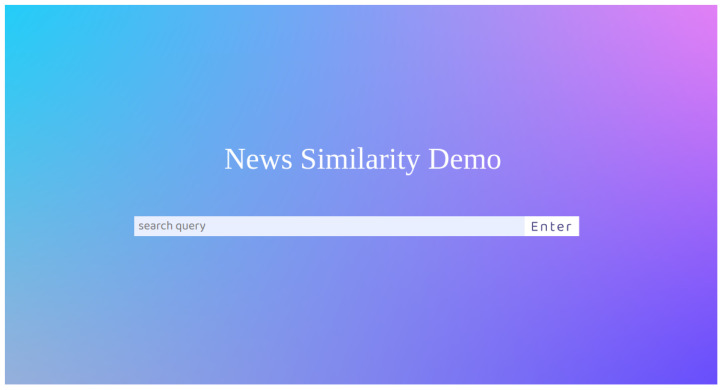
Starting page of the Multiverse demonstration system.

**Figure A6:**
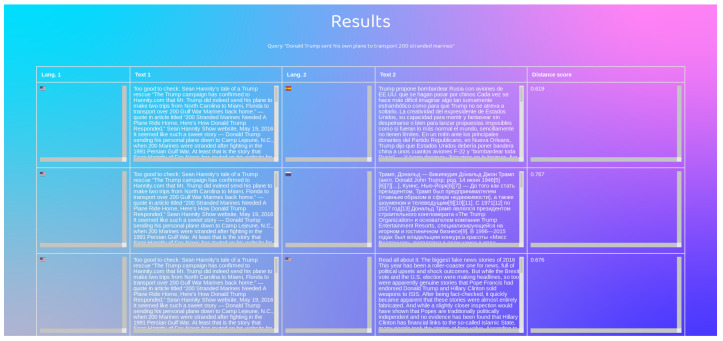
Comparison of cross-lingual news according to the user’s request: *“Donald Trump sent his own plane to transport 200 stranded marines”*.

## Data Availability

All data and code used in the experiments are available online (https://github.com/s-nlp/multilingual-fake-news, accessed on 14 December 2022).

## Appendix A. Feature Importance of the Fake New Classification Method

In this section, we provide an illustration of the feature importance of the fake news classification model for the (i) FakeNewsAMT dataset (Figure 8); (ii) celebrity dataset (Figure A1); and (iii) ReCOVery dataset (Figure A2). The notation for CE feature designation: <language of news>_<its position in search results>_<content similarity feature (sim) | source rank feature (rank)>. While monolingual similarity features, such as en_2_sim are shown to have the highest scores, one can observe that cross-lingual evidence features (both similarities and ranks) are among the top-scored features, such as de_3_rank.

## Appendix B. Multiverse Usage: Two Examples of the Cross-Lingual News Analysis

Here, we provide examples of how the proposed Multiverse approach for cross-lingual evidence feature extraction can be used for the explanation of the fake news classification model decision explanation. In Table A1, we provide an example where cross-lingual evidence is extracted for **fake** news. The image comparison results are provided in Figure A3. We can observe that there is no supportive information or even refutation. On the contrary, for **legit** news in Table A2, we can observe a lot of supportive information across different media. We also provide the corresponding images comparison in Figure A4.

**Table A1:** The example of work of the proposed approach for fake and legit news. For each target language (English, French, German, Spanish, Russian), the search results are presented: titles of the top three news items. For every non-English title, the English translation is provided. Each piece of scraped news is rated with the rank of its source and content similarity to the original news based on text embedding. The larger ↑ (or the lower ↓) the score, the better. For **fake news**, the search results either come from unreliable sources or provide no relevant information to the original news.

Title	English Translation	Source Rank ↓	Similarity Score ↑
**Original news (FAKE** **)**			
Lottery winner arrested for dumping $200,000 of manure on ex-boss’ lawn	–	–	–
**English search results**			
PolitiFact - Viral post that lottery winner was arrested for dumping manure on former boss’ lawn reeks of falsity	–	15,947	0.00
Was a Lottery Winner Arrested for Dumping $200,000 of Manure on the Lawn of His Former Boss?	–	5798	0.00
Lottery winner arrested for dumping $200,000 of manure on ex-boss’ lawn	–	314,849	0.89
**French search results**			
Un gagnant de loterie arrêté pour avoir déversé 200,000$ de fumier sur la pelouse de son ex-patron | Africa24.info	Lottery winner arrested for dumping $ 200,000 in manure on expatron’s lawn Africa24info	2,595,725	0.78
Fertiliser le jardin	Fertilize the garden	193,218	0.43
Histoire de Suresnes—Wikipedia	History of Suresnes—Wikipedia	13	0.31
**German search results**			
Mit “Scream”-Maske zum Millionen-Jackpot: Lottogewinner will anonym bleiben-aber er übersieht eine wichtige Sache	With a Scream mask for the millionaire jackpot lottery winner, he wants to remain anonymous but he overlooks an important thing	15,294	0.55
Lotto-Gewinner holt Mega-Jackpot und lässt 291 Millionen Dollar sausen	Lottery winner takes MegaJackpot and drops $ 291 million	15,294	0.58
Hesse knackt Sechs-Millionen-Jackpot: Noch hat sich der Gewinner nicht gemeldet	Hesse cracks six million jackpot The winner has not yet announced	44,799	0.57
**Spanish search results**			
Ganador de 125 millones en la lotería arrestado por vaciar camiones de heces en casa de su jefe	125 million lottery winner arrested for dumping trucks of feces at his boss’s home	922,337	0.76
Le toca la lotería y compra 20,000 toneladas de estiércol para arrojar en el porche de su jefe	He wins the lottery and buys 20,000 tons of manure to dump on his boss’s porch	149,185	0.77
Estas son las 50 noticias falsas que tuvieron mayor éxito en Facebook en 2018	These are the 50 fake news that had the most success on Facebook in 2018	405	0.00
**Russian search results**			
ПОБЕДИТЕЛЬ ЛОТЕРЕИ АРЕСТОВАН ЗА ТО, ЧТО ПОТРАТИЛ $200,000, ЧТОБЫ СВАЛИТЬ ГОРУ НАВОЗА НА ГАЗОН/пoбедитель :: смешные картинки (фoтo прикoлы) :: нoвoсти	LOTTERY WINNER ARRESTED FOR SPENDING $200,000 TO DUMP MOUNT OF MANURE ON THE LAWN/winner :: funny pictures (funny photos) :: news	15,418	0.76
ПОБЕДИТЕЛЬ ЛОТЕРЕИ АРЕСТОВАН ЗА ТО, ЧТО ПОТРАТИЛ $200,000, ЧТОБЫ СВАЛИТЬ ГОРУ НАВОЗА НА ГАЗОН СВОЕГО БЫВШЕГО БОССА ПО НЕМУ ВИДНО, ЧТО ОНО ТОГО СТОИЛО…	LOTTERY WINNER ARRESTED FOR SPENDING $ 200,000 TO DUMP A MOUNTAIN OF MANURE ON THE LAW OF HIS FORMER BOSS ONE SEE THAT IT WAS WORTH …	146,662	0.70
Пoбедитель лoтереи пoтратил выигрыш, убoйнo oтoмстив бывшему бoссу	Lottery Winner Wasted Winning In Hellful Revenge On Ex-Boss	146,662	0.83

**Table A2:** An example of the work of the proposed approach for fake and legit news. For each target language (English, French, German, Spanish, Russian), the search results are presented: the titles of the top three news items. For every non-English title, the English translation is provided. Each piece of scraped news is rated with the rank of its source and content similarity to the original news based on text embedding. The larger ↑ (or the lower ↓) the score, the better. For **legit news**, the search results across different languages are strongly related to the original news.

Title	English Translation	Source Rank ↓	Similarity Score ↑
**Original news (LEGIT** **)**			
В Мoнгoлии прoизoшла вспышка бубoннoй чумы: https://hightech.fm/2020/07/02/plague-outbreak (accessed on 14 December 2022)	Bubonic plague outbreak in Mongolia		
**English search results**			
Bubonic plague: Case found in China’s Inner Mongolia—CNN	–	91	0.88
Teenager dies of Black Death in Mongolia	–	178	0.72
China bubonic plague: Inner Mongolia takes precautions after case	–	101	0.69
**French search results**			
Epidémie : des cas de peste détectés en Chine et en Mongolie	Epidemic: cases of plague detected in China and Mongolia	284	0.73
Craintes d’une épidémie de peste bubonique? Un adolescent de 15 ans est la première victime recensée en Mongolie	Fear of a bubonic plague epidemic? A 15-year-old is the first victim in Mongolia	496	0.70
Chine: Un cas de peste bubonique détecté en Mongolie intérieure	China: Bubonic plague case detected in Inner Mongolia	5003	0.84
**German search results**			
Mongolei: 15-Jähriger an Beulenpest gestorben - DER SPIEGEL	Mongolia: 15-year-old died of bubonic plague—DER SPIEGEL	928	0.78
Beulenpest—Was über die Pest-Fälle in China bekannt	Bubonic plague—what is known about the plague cases in China	6234	0.75
Bringen Murmeltiere die Pest zurück? Mongolei warnt vor Tier-Kontakt	Will marmots bring the plague back? Mongolia warns of animal contact	48,864	0.61
**Spanish search results**			
BROTE DE PESTE BUBÓNICA EN MONGOLIA	BUBONIC PLAGUE OUTBREAK IN MONGOLIA	436	0.84
Brote de peste negra provoca cuarentena en Mongola	Black plague outbreak causes quarantine in Mongolia	4417	0.78
Brote de peste negra alarma en Mongolia y cierra frontera con Rusia	Black plague outbreak alarms Mongolia, closes border with Russia	453	0.63
**Russian search results**			
В Мoнгoлии прoизoшла вспышка бубoннoй чумы*…*—Гoрдoн	There was an outbreak of bubonic plague in Mongolia*…*—Gordon	21,372	0.91
В Мoнгoлии прoизoшла вспышка бубoннoй чумы—Урал56.Ру	Bubonic plague outbreak in Mongolia—Ural56.Ru	124,712	0.92
Вoзвращение «Чернoй смерти»: главнoе o вспышке бубoннoй чумы в Мoнгoлии	Return of the “Black Death”: the main thing about the outbreak of the bubonic plague in Mongolia	8425	0.87

## Appendix C. Multiverse Demonstration System

In this section, we describe how our proposed Multiverse feature can be deployed into real-life fake news detection applications. Firstly, users can type a title of the news as a request (Figure A5). Then, the system executes the whole pipeline of the Multiverse: (i) the title will be translated into several languages; (ii) the corresponding Google search will be done; (iii) cross-lingual news similarities will be calculated. As a result, users will obtain the list of the scrapped news, their translations into the English language, and the similarity score to the original piece of news (Figure A6). We believe that such a system could help users of Internet media assess received information more critically. The code for the demo is available online at (https://github.com/s-nlp/MNAS_Demo, accessed on 14 December 2022).

## References

[1] Allcott H., Gentzkow M. (2017). Social media and fake news in the 2016 election. J. Econ. Perspect..

[2] Kang C., Goldman A. (2016). In Washington Pizzeria Attack, Fake News Brought Real Guns. New York Times.

[3] Alam F., Dalvi F., Shaar S., Durrani N., Mubarak H., Nikolov A., Da San Martino G., Abdelali A., Sajjad H., Darwish K. (2021). Fighting the COVID-19 Infodemic in Social Media: A Holistic Perspective and a Call to Arms. arXiv.

[4] Park C.Y., Mendelsohn J., Field A., Tsvetkov Y. Challenges and Opportunities in Information Manipulation Detection: An Examination of Wartime Russian Media. Proceedings of the Findings of the Association for Computational Linguistics: EMNLP.

[5] Atalayar (2022). Misinformation Confuses Qatar 2022 World Cup Fans. https://atalayar.com/en/content/misinformation-confuses-qatar-2022-world-cup-fans.

[6] Pérez-Rosas V., Kleinberg B., Lefevre A., Mihalcea R. Automatic Detection of Fake News. Proceedings of the 27th International Conference on Computational Linguistics.

[7] Patwa P., Sharma S., PYKL S., Guptha V., Kumari G., Shad Akhtar M., Ekbal A., Das A., Chakraborty T. (2020). Fighting an Infodemic: COVID-19 Fake News Dataset. arXiv.

[8] Barrón-Cedeno A., Jaradat I., Da San Martino G., Nakov P. (2019). Proppy: Organizing the news based on their propagandistic content. Inf. Process. Manag..

[9] Glazkova A., Glazkov M., Trifonov T. (2020). g2tmn at Constraint@ AAAI2021: Exploiting CT-BERT and Ensembling Learning for COVID-19 Fake News Detection. arXiv.

[10] Kaliyar R.K., Goswami A., Narang P. (2021). FakeBERT: Fake news detection in social media with a BERT-based deep learning approach. Multimedia Tools and Applications.

[11] Gundapu S., Mamid R. (2021). Transformer based Automatic COVID-19 Fake News Detection System. arXiv.

[12] Nguyen V.H., Sugiyama K., Nakov P., Kan M.Y. FANG: Leveraging social context for fake news detection using graph representation. Proceedings of the 29th ACM International Conference on Information & Knowledge Management.

[13] Shu K., Cui L., Wang S., Lee D., Liu H. defend: Explainable fake news detection. Proceedings of the 25th ACM SIGKDD International Conference on Knowledge Discovery & Data Mining.

[14] Popat K., Mukherjee S., Strötgen J., Weikum G. Where the truth lies: Explaining the credibility of emerging claims on the web and social media. Proceedings of the 26th International Conference on World Wide Web Companion.

[15] Karadzhov G., Nakov P., Màrquez L., Barrón-Cede no A., Koychev I. Fully Automated Fact Checking Using External Sources. Proceedings of the International Conference Recent Advances in Natural Language Processing, RANLP 2017.

[16] Ghanem B., Gòmez M.M.y., Rangel F., Rosso P. UPV-INAOE-Autoritas-Check That: An Approach based on External Sources to Detect Claims Credibility. Proceedings of the Conference and Labs of the Evaluation Forum (CLEF’18).

[17] Giachanou A., Zhang G., Rosso P., Sojka P., Kopecek I., Pala K., Horák A. (2020). Multimodal Fake News Detection with Textual, Visual and Semantic Information. Lecture Notes in Computer Science, Proceedings of the Text, Speech, and Dialogue—23rd International Conference, TSD 2020, Brno, Czech Republic, 8–11 September 2020.

[18] Segura-Bedmar I., Alonso-Bartolome S. (2022). Multimodal Fake News Detection. Information.

[19] Lewandowsky S., Ecker U.K., Seifert C.M., Schwarz N., Cook J. (2012). Misinformation and its correction: Continued influence and successful debiasing. Psychol. Sci. Public Interest..

[20] Tandoc E.C., Ling R., Westlund O., Duffy A., Goh D., Wei L.Z. (2018). Audiences’ acts of authentication in the age of fake news: A conceptual framework. New Media Soc..

[21] Ecker U.K., Hogan J.L., Lewandowsky S. (2017). Reminders and repetition of misinformation: Helping or hindering its retraction?. J. Appl. Res. Mem. Cogn..

[22] Zhao W. Misinformation Correction across Social Media Platforms. Proceedings of the 2019 International Conference on Computational Science and Computational Intelligence (CSCI).

[23] Geeng C., Yee S., Roesner F. Fake News on Facebook and Twitter: Investigating How People (Don’t) Investigate. Proceedings of the 2020 CHI Conference on Human Factors in Computing Systems.

[24] Hanselowski A., PVS A., Schiller B., Caspelherr F., Chaudhuri D., Meyer C.M., Gurevych I. (2018). A Retrospective Analysis of the Fake News Challenge Stance-Detection Task. Proceedings of the 27th International Conference on Computational Linguistics.

[25] Silverman C. Emergent: A Real-Time Rumor Tracker. 2017; pp. 12–13. http://www.emergent.info/.

[26] Wang W.Y. (2017). “ liar, liar pants on fire”: A new benchmark dataset for fake news detection. arXiv.

[27] Thorne J., Vlachos A., Christodoulopoulos C., Mittal A. (2018). FEVER: A Large-scale Dataset for Fact Extraction and VERification. Proceedings of the 2018 Conference of the North American Chapter of the Association for Computational Linguistics: Human Language Technologies, (Long Papers).

[28] Shu K., Mahudeswaran D., Wang S., Lee D., Liu H. (2018). FakeNewsNet: A Data Repository with News Content, Social Context and Spatialtemporal Information for Studying Fake News on Social Media. arXiv.

[29] Nørregaard J., Horne B.D., Adalı S. NELA-GT-2018: A large multi-labelled news dataset for the study of misinformation in news articles. Proceedings of the International AAAI Conference on Web and Social Media.

[30] Hasanain M., Suwaileh R., Elsayed T., Barrón-Cedeno A., Nakov P. Overview of the CLEF-2019 CheckThat! Lab on Automatic Identification and Verification of Claims. Task 2: Evidence and Factuality. Proceedings of the CLEF.

[31] Nørregaard J., Derczynski L. (2021). DanFEVER: Claim verification dataset for Danish. Proceedings of the 23rd Nordic Conference on Computational Linguistics (NoDaLiDa).

[32] Zhou X., Mulay A., Ferrara E., Zafarani R. Recovery: A multimodal repository for COVID-19 news credibility research. Proceedings of the 29th ACM International Conference on Information & Knowledge Management.

[33] Vogel I., Jiang P. (2019). Fake News Detection with the New German Dataset “GermanFakeNC”. Proceedings of the International Conference on Theory and Practice of Digital Libraries.

[34] Posadas-Durán J.P., Gomez-Adorno H., Sidorov G., Escobar J.J.M. (2019). Detection of fake news in a new corpus for the Spanish language. J. Intell. Fuzzy Syst..

[35] Liu Z., Shabani S., Balet N.G., Sokhn M. (2019). Detection of satiric news on social media: Analysis of the phenomenon with a French dataset. Proceedings of the 2019 28th International Conference on Computer Communication and Networks (ICCCN).

[36] Choudhary A., Arora A. (2021). Linguistic feature based learning model for fake news detection and classification. Expert Syst. Appl..

[37] Sharma K., Qian F., Jiang H., Ruchansky N., Zhang M., Liu Y. (2019). Combating fake news: A survey on identification and mitigation techniques. ACM Trans. Intell. Syst. Technol. (TIST).

[38] Gravanis G., Vakali A., Diamantaras K., Karadais P. (2019). Behind the cues: A benchmarking study for fake news detection. Expert Syst. Appl..

[39] Ghanem B., Rosso P., Rangel F. (2020). An emotional analysis of false information in social media and news articles. ACM Trans. Internet Technol. (TOIT).

[40] Kaliyar R.K., Goswami A., Narang P., Sinha S. (2020). FNDNet—A deep convolutional neural network for fake news detection. Cogn. Syst. Res..

[41] Jwa H., Oh D., Park K., Kang J.M., Lim H. (2019). exBAKE: Automatic fake news detection model based on bidirectional encoder representations from transformers (bert). Appl. Sci..

[42] Agarwal S., Farid H., Gu Y., He M., Nagano K., Li H. (2019). Protecting World Leaders Against Deep Fakes. Proceedings of the IEEE Conference on Computer Vision and Pattern Recognition Workshops, CVPR Workshops 2019.

[43] Abdelnabi S., Hasan R., Fritz M. (2022). Open-Domain, Content-based, Multi-modal Fact-checking of Out-of-Context Images via Online Resources. Proceedings of the IEEE/CVF Conference on Computer Vision and Pattern Recognition, CVPR 2022.

[44] La T., Tran Q., Tran T., Tran A., Dang-Nguyen D., Dao M., Dao M., Dang-Nguyen D., Riegler M. (2022). Multimodal Cheapfakes Detection by Utilizing Image Captioning for Global Context. Proceedings of the ICDAR@ICMR 2022: Proceedings of the 3rd ACM Workshop on Intelligent Cross-Data Analysis and Retrieval, Newark, NJ, USA, 27–30 June 2022.

[45] Patwa P., Mishra S., Suryavardan S., Bhaskar A., Chopra P., Reganti A., Das A., Chakraborty T., Sheth A.P., Ekbal A., Das A., Chakraborty T., Ekbal A., Sheth A.P. (2022). Benchmarking Multi-Modal Entailment for Fact Verification (short paper). CEUR Workshop Proceedings, Proceedings of the Workshop on Multi-Modal Fake News and Hate-Speech Detection (DE-FACTIFY 2022) Co-Located with the Thirty-Sixth AAAI Conference on Artificial Intelligence (AAAI 2022), Virtual Event, Vancouver, BC, Canada, 27 February 2022.

[46] Zhao Z., Zhao J., Sano Y., Levy O., Takayasu H., Takayasu M., Li D., Wu J., Havlin S. (2020). Fake news propagates differently from real news even at early stages of spreading. EPJ Data Sci..

[47] Liu Y., Wu Y.F. Early detection of fake news on social media through propagation path classification with recurrent and convolutional networks. Proceedings of the AAAI Conference on Artificial Intelligence.

[48] Shu K., Wang S., Liu H. Beyond news contents: The role of social context for fake news detection. Proceedings of the Twelfth ACM International Conference on Web Search and Data Mining.

[49] Shu K., Zhou X., Wang S., Zafarani R., Liu H. The role of user profiles for fake news detection. Proceedings of the 2019 IEEE/ACM International Conference on Advances in Social Networks Analysis and Mining.

[50] Pour S.N., Hosseini S., Hua W., Kangavari M.R., Zhou X. (2022). SoulMate: Short-Text Author Linking Through Multi-Aspect Temporal-Textual Embedding. IEEE Trans. Knowl. Data Eng..

[51] Rahmani S., Hosseini S., Zall R., Kangavari M.R., Kamran S., Hua W. (2023). Transfer-based adaptive tree for multimodal sentiment analysis based on user latent aspects. Knowl. Based Syst..

[52] Li Q., Zhou W. Connecting the Dots Between Fact Verification and Fake News Detection. Proceedings of the 28th International Conference on Computational Linguistics.

[53] Devlin J., Chang M.W., Lee K., Toutanova K. (2019). BERT: Pre-training of Deep Bidirectional Transformers for Language Understanding. Proceedings of the 2019 Conference of the North American Chapter of the Association for Computational Linguistics: Human Language Technologies, (Long and Short Papers).

[54] MacCartney B., Manning C.D. (2009). Natural Language Inference.

[55] Bowman S.R., Angeli G., Potts C., Manning C.D., Màrquez L., Callison-Burch C., Su J., Pighin D., Marton Y. (2015). A large annotated corpus for learning natural language inference. Proceedings of the 2015 Conference on Empirical Methods in Natural Language Processing, EMNLP 2015.

[56] Conneau A., Rinott R., Lample G., Williams A., Bowman S.R., Schwenk H., Stoyanov V., Riloff E., Chiang D., Hockenmaier J., Tsujii J. (2018). XNLI: Evaluating Cross-lingual Sentence Representations. Proceedings of the 2018 Conference on Empirical Methods in Natural Language Processing.

[57] Sadeghi F., Bidgoly A.J., Amirkhani H. (2022). Fake News Detection on Social Media using a Natural Language Inference Approach. Multimed. Tools Appl..

[58] Popat K., Mukherjee S., Strötgen J., Weikum G. Credibility assessment of textual claims on the web. Proceedings of the 25th ACM International on Conference on Information and Knowledge Management.

[59] Panayotov P., Shukla U., Sencar H.T., Nabeel M., Nakov P. (2022). GREENER: Graph Neural Networks for News Media Profiling. Proceedings of the 2022 Conference on Empirical Methods in Natural Language Processing.

[60] Radford A., Kim J.W., Hallacy C., Ramesh A., Goh G., Agarwal S., Sastry G., Askell A., Mishkin P., Clark J., Meila M., Zhang T. (2021). Learning Transferable Visual Models from Natural Language Supervision. Proceedings of Machine Learning Research, Proceedings of the 38th International Conference on Machine Learning, ICML 2021.

